# Longitudinal Characterization of Giant *ANK2*-Depleted Monkeys Suggests Neurodevelopmental-Disorder-Like Phenotypes

**DOI:** 10.34133/research.1424

**Published:** 2026-09-07

**Authors:** Hong-Di Huang, Yun-Chao Ji, Long Zhang, Min Xu, Yicheng Qiao, Chen-Yao Li, Hui-Hen Xie, Yu Li, Bao-Lin Zhang, Ming-Hao Qiu, Ya-Li Zhang, Xiao-Mei Yu, Yun-Bing Zhang, Yun Wang, Qiong Wang, Yi-Jiang Li, Long-Bao Lv, Xing-Xu Huang, Bing-Yu Mao, Dong-Dong Wu, Ping Zheng, Ning Liu, Jian-Hong Wang, Yong-Gang Yao

**Affiliations:** ^1^ State Key Laboratory of Genetic Evolution and Animal Models, Yunnan Key Laboratory of Animal Models and Human Disease Mechanisms, and Yunnan Engineering Center on Brain Disease Models, Kunming, Yunnan 650204, China.; ^2^ National Research Facility for Phenotypic & Genetic Analysis of Model Animals (Primate Facility) and National Resource Center for Non-Human Primates, Kunming Institute of Zoology, Chinese Academy of Sciences, Kunming, Yunnan 650107, China.; ^3^ Kunming College of Life Sciences, University of Chinese Academy of Sciences, Kunming, Yunnan 650204, China.; ^4^ State Key Laboratory of Cognitive Science and Mental Health, Institute of Biophysics, Chinese Academy of Sciences, Beijing 100101, China.; ^5^ College of Life Sciences, University of Chinese Academy of Sciences, Beijing 101408, China.; ^6^ Yunnan Key Laboratory of Biodiversity Information, Kunming Institute of Zoology, Chinese Academy of Sciences, Kunming, Yunnan 650201, China.; ^7^ School of Life Science and Technology, Shanghai Tech University, Shanghai, China.; ^8^ KIZ/CUHK Joint Laboratory of Bioresources and Molecular Research in Common Diseases, Kunming Institute of Zoology, Chinese Academy of Sciences, Kunming, Yunnan 650204, China.

## Abstract

The *ANK2* gene mutations are marked risk factors for autism spectrum disorder, one of the neurodevelopmental disorders (NDDs) that often extends into adulthood and has a complex etiology involving genetic and environmental factors. *ANK2* encodes 2 major isoforms, AnkB-220 (220-kDa isoform of ankyrin-B) and giant AnkB-440. We previously generated a targeted knockout (KO) of giant AnkB-440 in 2 cynomolgus and 2 rhesus monkeys. While no autism-spectrum-disorder-like phenotypes were observed during infancy, we found marked brain volume loss. In this study, we conducted a longitudinal multimodal study in these giant *ANK2* KO monkeys during adolescent and young adulthood. Behavioral results from these giant *ANK2* KO monkeys revealed increased locomotor activity, deficient cognition (including working memory, cognitive flexibility, and operant lever-press learning), and impaired emotional regulation and social interaction. Furthermore, the giant *ANK2* KO monkeys exhibited persistent structural and functional brain abnormalities, up-regulation of brain triglyceride and glycerophospholipids, and peripheral blood transcriptome signatures of immune dysregulation, which may be related to their behavioral alternations. These observations suggest that giant *ANK2* depletion in non-human primates phenocopies aspects of behavioral, neural, and molecular features characteristic of NDDs. This study provides an in-depth exploratory longitudinal characterization of giant *ANK2* functions in primate brains, which may contribute to translational research on NDDs.

## Introduction

Human neurodevelopment is a prolonged process extending into adulthood. Abnormalities during this period can lead to neurodevelopmental disorders (NDDs), conditions that originate in development and result in deficits in personal and social functioning [1–3]. Common NDDs include autism spectrum disorder (ASD), intellectual disability (ID), attention-deficit/hyperactivity disorder, and motor disorders. Schizophrenia and neurological malformations are also proposed to belong to this spectrum [1–3]. The etiology of NDDs is complex, involving genetic and environmental factors [4–6].

The *ANK2* gene is crucial for neural development, playing a role in axonal guidance, neural circuit assembly [7–12], and stability of inhibitory synapses [13]. It encodes AnkB (ankyrin-B), which has 2 major isoforms: The widely expressed 220-kDa protein (AnkB-220) is found to function in dendritic spines, and the neuron-specific 440-kDa protein (giant AnkB-440) predominates in unmyelinated axons [12,14]. AnkB mutations are linked to peripheral pathologies, such as cardiac dysfunction, epilepsy, and metabolic syndrome [15–17]. Importantly, de novo *ANK2* mutations are marked risk factors for ASD [18–21] and are enriched in NDD populations presenting with developmental delay and ID [22–24], supporting *ANK2’*s associations with broader NDDs [22]. The giant ANK2 isoform (AnkB-440) encompasses the full AnkB-220 sequence along with additional exons, suggesting that mutations in AnkB-220 could also impact the giant ANK2 isoform. To date, however, researches have focused almost exclusively on mutations within the AnkB-220 sequence, leaving the functions of giant *ANK2* in the nervous system largely unexplored.

Previous studies using rodents revealed AnkB’s role in spine regulation, neuritogenesis, and axonal transport and development [10,12,14]. *ANK2* knockout (KO) mice showed disrupted neurogenesis and neuronal migration, axon tract loss, enlarged lateral ventricles [7,25,26]. Mice with a mutation or deficiency in giant AnkB-440 exhibited ASD-like behaviors and aberrant structural connectivity probably due to the gain of axon branching [10]. However, translating findings from mice to humans is challenging because of significant interspecies differences in genetics and neurobiology [27]. For example, *SHANK3* or *PINK1* mutant non-human primates (NHPs) exhibited neuronal development disruptions not seen in mice [28,29], highlighting the need for NHPs in neurological research, which share the closest phylogenetic relationship to humans and display similar brain developmental trajectories and architectural features [30–33]. Furthermore, the symptoms of NDDs typically emerge and reach full manifestation during late childhood or adolescence, a developmental period that is markedly compressed in rodents yet significantly extended in primates. Consequently, longitudinal studies in NHPs are indispensable for accurately delineating the complete disease course.

Previously, we generated a targeted KO of giant ANK2 (AnkB-440) in rhesus monkeys and cynomolgus monkeys [11] to better model human ASD or other NDDs. Surprisingly, we observed pronounced brain volume loss in these KO monkeys. However, behavioral assessments in infancy revealed no significant differences in social interaction, exploratory or stereotypical behaviors, sleep, or activity levels between KO and control monkeys. These phenotypes were dramatically different from those of mice [10]. Although early brain structural abnormalities are clearly evident, the questions of particular relevance in NHPs remain unknown: whether these deficits persist or progressively worsen over time and how giant *ANK2* depletion affects the brain during adolescence and young adulthood.

Guided by our infantile observations, we speculated that giant *ANK2* KO would drive a suite of behavioral, neural, and molecular alterations that unfold across adolescence and into adulthood. To address this, we performed a longitudinal multimodal characterization of previously generated giant AnkB-440 KO monkeys, tracking them from adolescence through young adulthood (Fig. S1). Our analyses revealed heightened activity levels without stereotypical behaviors, multiple cognitive deficits including impairments in working memory, cognitive flexibility, and operant lever-press learning, as well as persistent structural and functional brain abnormalities. Notably, these mutant animals exhibited pronounced social interaction deficits, emotional dysregulation, and abnormalities in both metabolomic and blood transcriptomic profiles.

Collectively, these findings substantiate the involvement of giant *ANK2* in critical neurofunctional domains and lay a potential platform for unraveling the cross-species mechanisms of giant *ANK2*-related neurological disorders, as well as developing possible therapeutic interventions in primates.

## Results

During a 7-year follow-up study, we performed a battery of behavioral tests to assess general symptoms, including neurological function, sleep patterns, exploratory behaviors, and manual dexterity, on the KO monkeys and age-matched wild-type (WT) monkeys (Fig. S1). Partly consistent with our previous observations for these monkeys from infancy to 2 years old [11], we found no significant differences between the 2 monkey groups at adolescent and young adulthood in these paradigms (Fig. S2A to E).

### The giant *ANK2* KO monkeys displayed distinct posture and hyperactivity

**Fig. 1.**
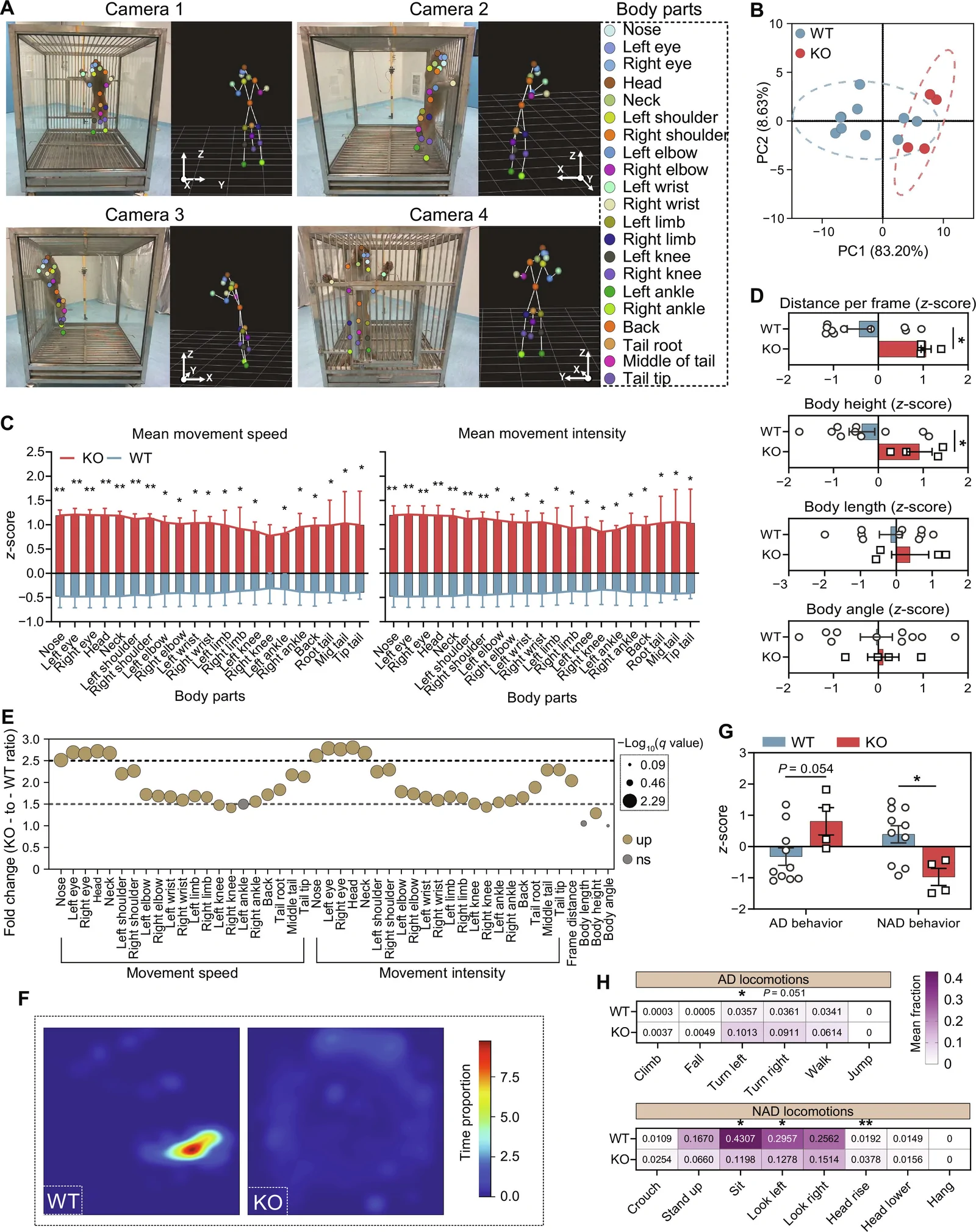
Immoderate activity and aberrant free-moving behaviors in the giant *ANK2* knockout (KO) rhesus monkeys. (A) Schematic diagram of animal recording using 4 synchronous cameras and the reconstructed skeletons (shown right of the camera pictures) using a well-trained model. The model can precisely recognize 21 body parts (dashed box). (B) Principal components analysis (PCA) score plot for monkeys based on 46 kinematic parameters. (C) Bar plot comparing the mean movement speed and movement energy of each labeled body part showed increased activity in KO monkeys compared to wild-type (WT) monkeys. (D) Bar plot comparing the mean movement distance per frame, mean body height, mean body length, and mean body angle between KO and WT groups. (E) Fold change calculated by the mean of KO to WT ratio for each kinematic parameter. Brown dots denoted significant up-regulation (up), while gray dots denoted no significant (ns) difference. Size of the dot denoted −log_10_ (*q* value). *q* value = *P* value corrected by false discovery rate (FDR). (F) Representative location heatmap of WT and KO monkeys. Color bar presenting the proportion of time spent. (G) Bar plot comparing the apparent displacement (AD) and non-AD (NAD) behaviors showed different body postures and general behavior spectrum between KO and WT groups. (H) Heatmap displaying the mean proportion of AD and NAD behavior. Color bars and figures in the cell presented the mean proportions of each group. *P < 0.05 and **P < 0.01, KO versus WT; multiple *t* tests with FDR correction for (C) and (D); Student’s *t* tests or Mann–Whitney *U* test for (G) and (H). Data were presented as means ± SEM. Rhesus KO group, *n* = 2; rhesus WT group, *n* = 5.

We adopted an artificial-intelligence-based 3-dimensional (3D) motion recognition framework [34,35] to estimate changes in free-moving behaviors and posture, which might decode the monkey’s behavioral sequence with higher granularity in both temporal (1/30 s, 30 fps) and spatial (millimeter-scale) levels (Fig. 1A). Under this framework, videos captured from a precalibrated multiview sampling system were conducted to automatically label and track 3D coordinates of 21 key body parts by a well-trained deep learning model for rhesus monkeys (Fig. 1A) [35]. The model precisely recognized the body parts and reconstructed the 3D skeletons matching the original postures, which ensured the reliability of the system and data for downstream analysis (Fig. 1A) and was used only for rhesus monkeys in this study.

In the subsequent investigation, we observed that the 2 KO rhesus monkeys could be clearly separated from 5 WT rhesus monkeys in the 2D space in the scores plot of principal components analysis (PCA) on the basis of extracted kinematic parameters (Fig. 1B). Group-by-group comparison illustrated that 43 of 46 parameters were significantly increased in the KO rhesus monkeys, including the moving speed and moving intensity of most of labeled body parts, as well as the moving distance per frame and body height (Fig. 1C and D and Table S1; e.g., moving speed of right knee, corrected *P* = 0.068; moving speed and intensity of other parts, corrected *P* < 0.05; distance, corrected *P* = 0.014; height, corrected *P* = 0.027). However, KO rhesus monkeys exhibited similar body length and body angle to those of the WT monkeys (Fig. 1D). We calculated the fold change of the KO rhesus monkeys as the ratio of KO to WT. Nearly half of the kinematic parameters that differed significantly were 2-fold higher in KO monkeys than in WT monkeys (e.g., movement speeds of head and shoulder regions), while the remaining parameters were approximately 1.5-fold higher (Fig. 1E). These data indicated that the KO rhesus monkeys exhibited stronger motional amplitude.

According to the position information in 3D coordinates, the location preference was determined. The location graph mapping the time proportion spent in any location by the monkeys demonstrated that the KO rhesus monkeys had no obvious position preference. In contrast, the WT rhesus monkey spent more time on a specific point, although each individual was heterogeneous, shown as the location heatmap (Fig. 1F). We analyzed 14 locomotor behaviors and categorized them into apparent displacement (AD) behaviors and non-AD (NAD) behaviors. The AD behaviors included climbing, falling, turning left/right, walking, and jumping, while the NAD behaviors included crouching, standing up, sitting, looking left/right, head rising/lowering, and hanging (Table S2). The KO rhesus monkeys displayed higher proportions in AD behaviors and lower proportions in NAD behaviors (Fig. 1G, AD: *P* = 0.054 and NAD: *P* = 0.014). Specifically, a higher movement fraction on turning left (*P* = 0.036) and turning right (*P* = 0.051) and a lower movement fraction on sitting (*P* = 0.020) and looking left (*P* = 0.036) were detected in KO rhesus monkeys (Fig. 1H). However, it exhibited significantly more head rising than the WT monkeys (Fig. 1H, *P* = 0.002). This behavior characteristic paralleled with the location pattern. We did not find significantly higher stereotypical behaviors in KO monkeys, resembling the infantile observations [11].

Collectively, these results indicated that KO rhesus monkeys exhibited increased activity, altered body postures, and a broad spectrum of general behavioral changes in the same novel environment.

### Cognitive function was impaired in the giant *ANK2* KO monkeys

**Fig. 2.**
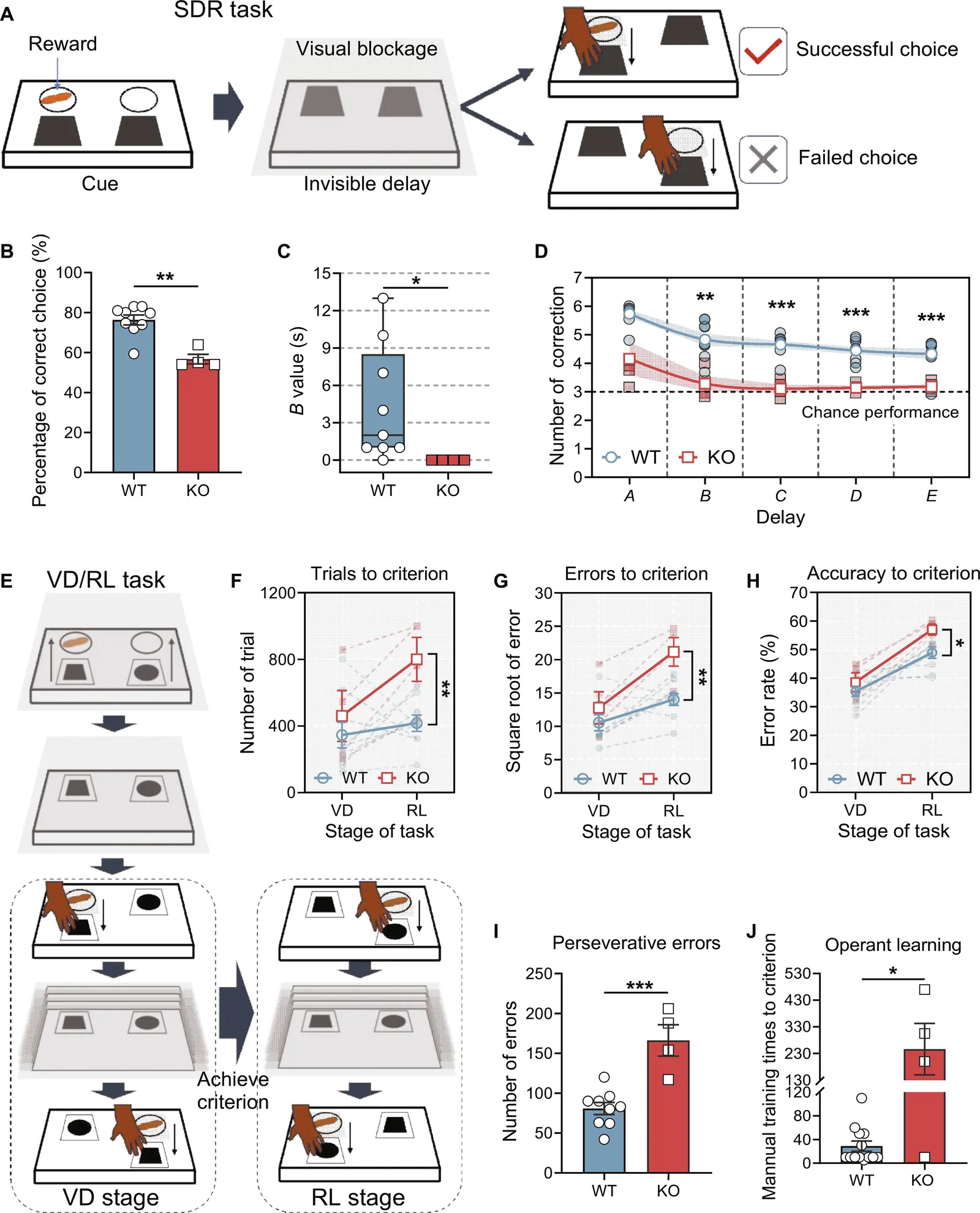
Impairment of cognitive functions in the giant *ANK2* knockout (KO) monkeys. (A) Schematic diagram of spatial delayed response (SDR) task using a Wisconsin general test apparatus. (B to D) Monkey performance in working memory assessed by the SDR task. The KO monkeys showed a lower percentage of correct choice when *B* = 2 s (B), lower *B* delay (C), and less mean correctness across 5 delays (D). (E) Schematic diagram of visual discrimination and reversal learning (VD/RL) task using a Wisconsin general test apparatus. (F to I) Monkey performance in cognitive flexibility assessed by the VD/RL task. In the stimulus discrimination stage, KO monkeys had similar numbers of training trials (F), error numbers (G) and mean error rate (H) as the WT controls, indicating normal associative learning. However, they showed impaired cognitive flexibility in the RL task, requiring more training trials (F), making more errors (G and I), and achieving lower accuracy (H). (J) Monkey performance in the operant learning (OL) task. The KO monkeys had more manual training trials to reach criterion, indicating defects in OL. *P < 0.05, **P < 0.01, and ***P < 0.001, KO versus WT; Mann–Whitney *U* test for (B) and (J); one-sample *t* test for (C); linear mixed-effects model (LMM) with Fisher’s least significant difference (LSD) multiple comparisons for (D) to (H); Student’s *t* tests for (I). Data were presented as means ± SEM. KO group, *n* = 4 (including 2 rhesus and 2 cynomolgus monkeys); WT group, *n* = 9 for (B) to (I) (3 rhesus and 6 cynomolgus monkeys) and *n* = 13 for (J) (10 rhesus and 3 cynomolgus monkeys).

As species-specific analysis showed no differences between rhesus and cynomolgus monkeys in performance on the cognitive tasks (Fig. S3), we combined data from both species to maximize the sample size within each experimental group. In the spatial delayed response (SDR) task (Fig. 2A), 4 KO monkeys (from both species) exhibited impaired working memory. In detail, they failed to reach the criterion of 86.7% correct responses when the delay *B* value exceeded 0 s. The KO monkeys presented a significantly lower percentage of correct responses at *B* = 2 s (Fig. 2B, *P* = 0.006) and also showed a significantly lower maximum *B* value than the 9 WT monkeys (Fig. 2C, *P* = 0.023). We also compared the average accuracy across all trials with *B* > 0 s using linear mixed-effects model (LMM). The significant fixed effect of delay (*P* < 0.001) revealed that accuracy decreased as the delay increased in both groups (Fig. 2D). In addition, a fixed effect of group was observed, indicating that the KO group performed worse than the WT group across all 5 delays: delay *A* (0 s, *P* = 0.070), delay *B* (*P* = 0.003), delay *C* (*P* < 0.001), delay *D* (*P* < 0.001), and delay *E* (*P* < 0.001) (Fig. 2D).

In the visual discrimination and reversal learning (VD/RL) task (Fig. 2E), the 4 KO monkeys presented normal association learning but insufficient cognitive flexibility. LMM revealed no differences in task stage × genotype interactions for trials to criteria (Fig. 2F) and errors to criterion (Fig. 2G), indicating that the pattern of task-related changes in trials or errors to criterion was comparable across groups. There were significant fixed effects of task stage (trials: *P* = 0.027 and errors: *P* < 0.001) and genotype (trials: *P* = 0.040 and errors: *P* = 0.023) on trials and errors before achieving criteria (Fig. 2F and G). Although post hoc comparisons by Fisher’s least significant difference (LSD) test showed no differences in the number of trials or errors during the VD stage, the KO group performed markedly more trials (Fig. 2F, *P* = 0.009) and errors (Fig. 2G, *P* = 0.003) to criteria than the WT monkeys when the stimulus-reward association was reversed in the RL stage. Likewise, KO monkeys had similar mean error rate relative to WT monkeys in the VD stage, whereas the mean error rate of KO monkeys was significantly higher than that of the WT monkeys in the RL stage (Fig. 2H, *P* = 0.026). A trial-by-trial analysis demonstrated that KO monkeys made 2 times more perseverative errors to achieve the criteria after reversal (Fig. 2I, *P* < 0.001). In addition, the 4 KO monkeys showed significantly lower performance on the operant learning (OL) task compared to eleven WT monkeys, as reflected by the need for more experimenter-guided/mannual training trials to reach criterion in KO monkeys (Fig. 2J, *P* = 0.044). This result suggested that the KO monkeys had impaired ability for OL. Taken together, KO of giant *ANK2* in both species (rhesus and cynomolgus) might contribute the underlying cause of cognitive deficits in adolescent monkeys.

### Abnormalities of emotional regulation and social interaction in giant *ANK2* KO monkeys

**Fig. 3.**
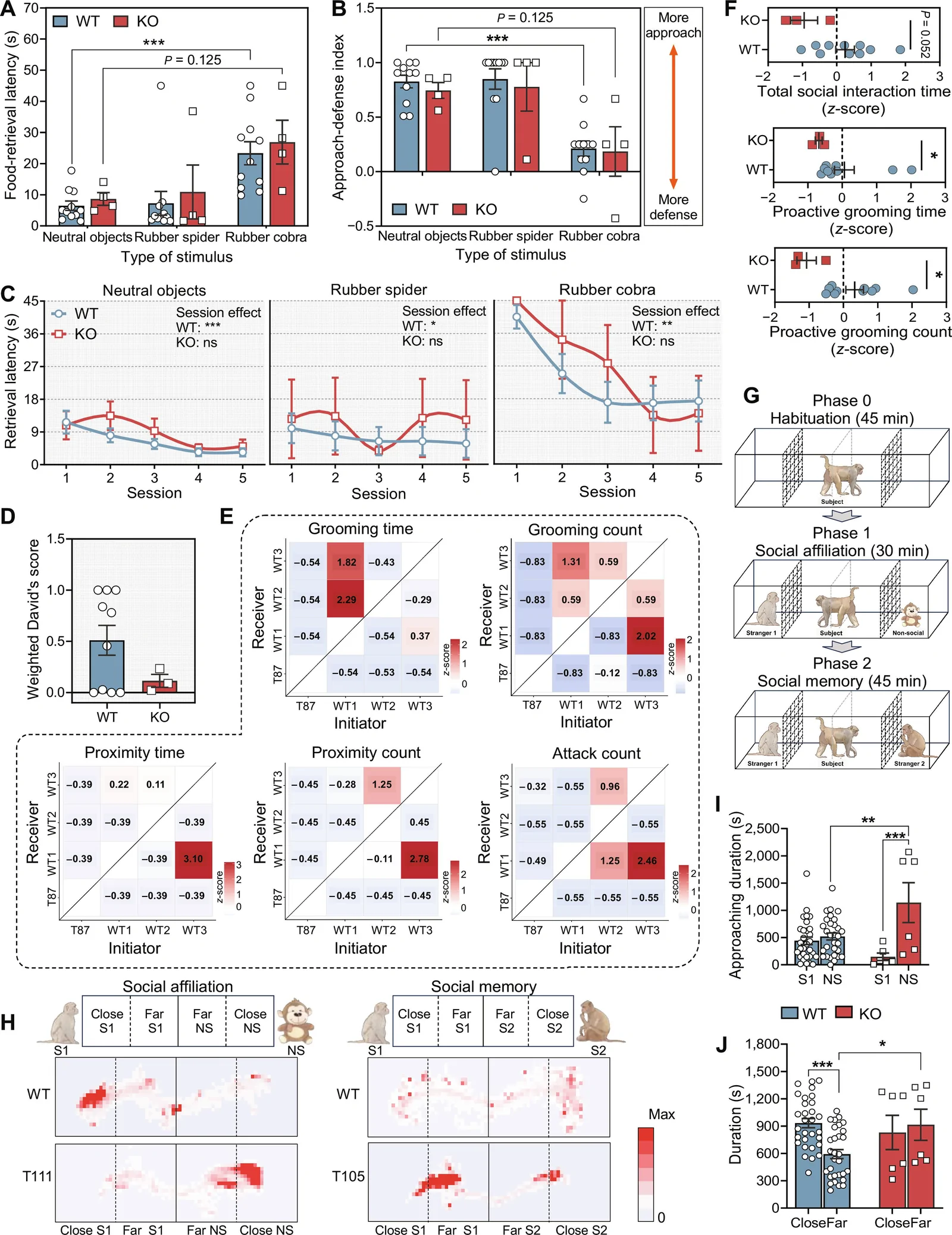
Abnormal emotional responses and social interaction in the giant *ANK2* knockout (KO) monkeys. (A) Average food-retrieval latency in the emotional response (ER) test. The wild-type (WT) monkeys, but not KO monkeys, displayed significantly longer latency to retrieve food when facing a cobra toy than neutral objects. (B) Behaviors during the ER test scored as the approach-defense index. The WT monkeys, but not KO monkeys, had significantly lower scores when facing a cobra toy than neutral objects. (C) Line plots showing the average food-retrieval latency for each stimulus across 5 sessions. The 2 groups exhibited similar retrieval latency. Unlike KO monkeys, WT monkeys showed significant habituation, with latency decreasing across sessions. (D) Weighed David’s score showing the lower tendency of social hierarchy of KO monkeys (with the exclusion of T105) compared to WT monkeys. (E) Heatmaps of *z*-score normalized dyadic grooming time and count, proximity time and count, and attack count among individual monkeys in one colony. The *z*-score between a pair of initiator and receiver was defined by a color (red, above the group mean; blue, below the group mean; white, near the group mean). (F) Social behaviors in group-rearing conditions. The KO monkeys showed a lower tendency for total social interaction time and significantly lower proactive grooming time and grooming frequency. (G) Schematic of the 3-chamber social interaction test (SIT) in rhesus monkeys, showing habituation (phase 0), social affiliation (phase 1), and social memory (phase 2). (H) Heatmaps of monkeys approaching duration to stranger 1 (S1)/nonsocial object (NS) in phase 1, and S1/stranger 2 (S2) in phase 2 from the lateral view. (I and J) Quantification of 3-chamber SIT. The KO monkeys preferentially approached the NS object but not S1 in phase 1 (I). The KO monkeys showed increased avoidance (far) of conspecifics relative to WT monkeys in phase 2 (J). *P < 0.05, **P < 0.01, and ***P < 0.001, KO versus WT; Wilcoxon matched-pairs signed-rank test for (A) and (B); Friedman test for (C); Student’s *t* tests or Mann–Whitney *U* test for (D) and (F); LMM with Fisher’s LSD multiple comparisons for (I) and (J). Data were presented as means ± SEM, except each trial for individual monkey for (I) and (J). The animal numbers varied in (A) to (C): KO, *n* = 4 (including 2 rhesus and 2 cynomolgus monkeys); WT, *n* = 11 (including 5 rhesus and 6 cynomolgus monkeys); (D) to (F): KO, *n* = 3 (including 1 rhesus and 2 cynomolgus monkeys; one KO rhesus monkey [T105] was excluded); WT, *n* = 10 (including 2 rhesus and 8 cynomolgus monkeys); and (G) to (J): KO, *n* = 2 rhesus monkeys; WT, *n* = 10 rhesus monkeys.

To test whether the giant *ANK2* KO would impact the emotion and social interaction of monkeys, we performed emotional response (ER) tests and social interaction tests (SITs) on the monkeys. The mean retrieval latency across 5 sessions showed that the WT group displayed a significantly increased latency when neutral objects were shifted to the fear-inducing rubber cobra (*P* = 0.001), whereas no significant difference was found in the KO group, albeit there was a tendency for increased latency (*P* = 0.125) (Fig. 3A). Consistently, the approach-defense index calculated from behavioral scores also showed a significantly increased defensive behavior of WT monkeys (*P* = 0.001) but not of KO monkeys (*P* = 0.125) (Fig. 3B). These findings indicated that the KO group exhibited altered emotional responses to fearful stimuli and failed to inhibit their food retrieval responses. Note that the KO group had a small number of animals and the values should be interpreted with caution.

We next assessed the latency across the 5 sessions. A group-by-group comparison exhibited that the KO and WT groups had similar food-retrieval latencies in each session regardless of whether it was a neutral or negative condition (Fig. 3C). However, a within-group analysis revealed that the WT monkeys would adapt to the fearful objects, evidenced by a significant reduction of food-retrieval latency in the presence of the 3 stimuli as the testing sessions progressed (Fig. 3C, neutral: *P* < 0.001, spider: *P* = 0.011, and snake: *P* = 0.002). On the other hand, KO monkeys did not exhibit such an adaptation (Fig. 3C). Moreover, the 2 groups showed no difference in latency of neutral trials immediately before and after the snake emerged (Fig. S2E), indicating that the designed order in which negative stimuli were presented was not a factor affecting the results.

The observation from playback of behavioral records under the group-rearing condition showed that the KO monkeys presented a solitary trait (Fig. S2F) and the monkey T105 preferred to stay in the inner room rather than the outside room in the single colony unit (Fig. S2G). Quantitative analysis of the social behaviors showed that the 3 KO monkeys (from 2 species, excluding T105) were scored with a tendency toward lower social hierarchy (Fig. 3D) and lower social interaction time (Fig. 3E and F, *P* = 0.052). Moreover, they also exhibited significantly lower proactive grooming time (*P* = 0.018) and proactive grooming count (Fig. 3E and F, *P* = 0.018). However, no differences were found in the duration of passive grooming, passive social behaviors, and social withdrawal (Fig. S2H). Consequently, the social interaction deficit appeared specific to proactive behaviors. To further confirm this observation, we performed the 3-chamber SIT in rhesus monkeys (Fig. 3G). In the social affiliation test (phase 1), the 2 KO rhesus monkeys spent significantly more time approaching the nonsocial object (toy monkey) than stranger 1 (*P* < 0.001), and this nonsocial approaching duration was also significantly prolonged compared with that of WT monkeys (Fig. 3H and I, *P* = 0.001). In the social memory test (phase 2), KO monkeys displayed increased social avoidance, spending significantly longer time distant from both strangers 1 and 2, relative to WT monkeys (Fig. 3H and J, *P* = 0.021). No significant difference was observed between approach and avoidance durations toward the 2 conspecifics in KO monkeys, whereas WT monkeys exhibited significantly longer approach than avoidance durations toward both strangers (Fig. 3J, *P* < 0.001). Together, the results suggested that KO monkeys prefer nonsocial objects over conspecifics, which may explain their diminished proactive behaviors in the group.

### MRI-based assessment of brain structural and functional changes in giant *ANK2* KO monkeys

**Fig. 4.**
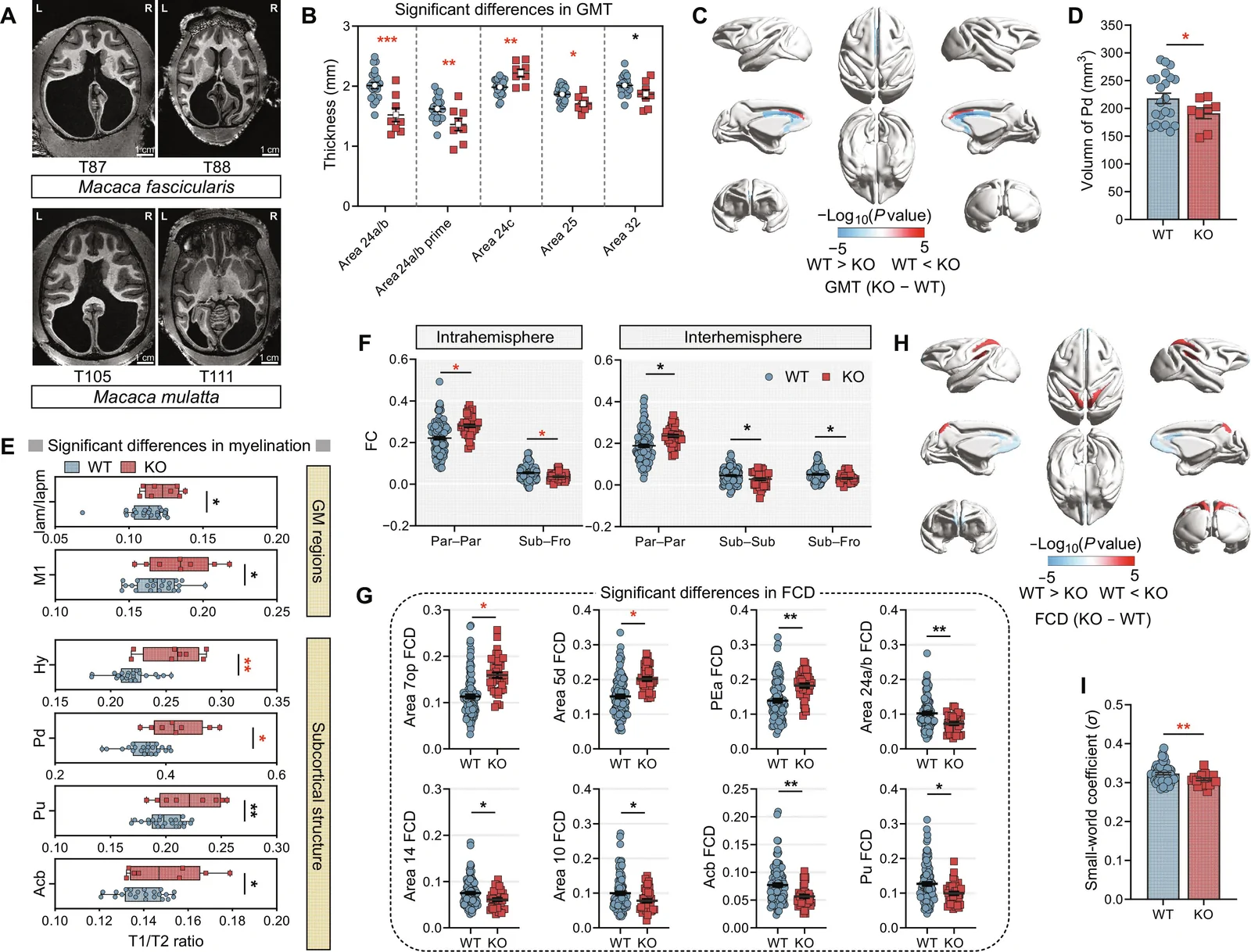
Abnormal brain structures and functional connectivity (FC) in the giant *ANK2* knockout (KO) monkeys. (A) Representative axial slices of T1-weighted images showing morphological alterations in brain structure for each KO monkey at 4 years old. Please note that the MRI images of KO monkeys T88 and T105 were incorrectly switched in the original Fig. S5 of our earlier publication (Qin et al. [11]). (B) Regions in the frontal and parietal lobes exhibiting differences in gray matter thickness (GMT) between KO and wild-type (WT) monkeys. (C) Mapping of GMT altered regions in (B) onto a monkey brain template. (D) Significant volume difference of the pallidum (Pd) between KO and WT monkeys. (E) Regions showing differences in myelination, calculated from the T1-weighted/T2-weighted ratio between KO and WT monkeys. GM, gray matter; Hy, hypothalamus; Pu, putamen; Acb, accumbens. (F) Lobe pairs showing differences in intra- and interhemispheric FC between KO and WT monkeys. (G) Regions exhibiting differences in FC density (FCD) between KO and WT monkeys. (H) Mapping of altered regions in (H) onto a monkey brain template. (I) Small-world sigma coefficient, showing significant difference between WT and KO monkeys. Regions/lobe pairs surviving false discovery rate (FDR) correction and regions/lobe pairs showing group differences at the uncorrected threshold are displayed. *P < 0.05, **P < 0.01, and ***P < 0.001, KO versus WT; generalized linear-mixed models. Red asterisks denote FDR-corrected *P* values; black asterisks denote uncorrected *P* values. In the box plots of (E), the center line represents the median, the box indicates the interquartile range, and the whiskers extend to 1.5 times the interquartile range to illustrate the full data distribution. Other data were presented as means ± SEM. KO group, *n* = 4 (including 2 rhesus and 2 cynomolgus monkeys); WT group, *n* = 11 (including 5 rhesus and 6 cynomolgus monkeys). The brain regions were defined in Table S3.

Consistent with our previous observation of drastic brain structural change in these KO monkeys at 6, 12, and 24 months of age [11], we found sustained cerebral ventricular enlargement in KO monkeys by 4 years of age, leading to pronounced morphological alterations in the occipital, temporal, and posterior parietal lobes (Fig. 4A). To minimize segmentation errors resulting from these anatomical alterations, we restricted the structural and functional MRI analysis to the frontal lobe (29 regions), the anterior parietal lobe (10 regions), and 6 subcortical regions (Table S3 and Fig. S4).

We first examined cortical gray matter thickness (GMT). KO monkeys of both species showed significantly reduced GMT in 3 frontal regions (area 24a/b: corrected *P* < 0.001, area 24a/b prime: corrected *P* = 0.007, and area 25: corrected *P* = 0.033) compared to WT monkeys. At the uncorrected threshold, area 32 also showed reduced GMT (*P* = 0.019) in KO monkeys (Fig. 4B). In contrast, area 24c showed significantly increased GMT in KO monkeys relative to WT monkeys (corrected *P* = 0.007) (Fig. 4B). No significant differences were observed in parietal regions, either before or after correction. Overall, these GMT analyses demonstrated altered frontal cortical gray matter in KO monkeys, with most false discovery rate (FDR)-corrected regions showing reduced thickness (Fig. 4B and C). To further characterize the changes in gray matter morphology, we analyzed cortical surface area and gray matter volume. These analyses identified several regions showing group differences at the uncorrected threshold, with most regions showing reduced values in KO monkeys (Fig. S5A to D). Among the subcortical regions examined, pallidum volume was significantly reduced in KO monkeys (Fig. 4D, corrected *P* = 0.030). We also examined T1-weighted/T2-weighted-based myelination estimates. KO monkeys exhibited significantly increased myelination in 2 subcortical structures (Fig. 4E, hypothalamus: corrected *P* = 0.004 and pallidum: corrected *P* = 0.019). Additional increases were observed at the uncorrected threshold in the accumbens (*P* = 0.036), putamen (*P* = 0.008), M1 (*P* = 0.036), and medial agranular insular area (lam)/posteromedial agranular insular area (lapm) (*P* = 0.040), although these did not reach FDR-corrected significance.

Next, we analyzed functional connectivity (FC) based on resting-state functional MRI (rs-fMRI) data using generalized LMMs at both the lobar and regional levels. At the lobar level, KO monkeys exhibited altered intra- and interhemispheric FC patterns (Fig. 4F). Specifically, intrahemispheric FC within the parietal lobe was significantly increased (corrected *P* = 0.025), while intrahemispheric FC between subcortical structures and the frontal lobe was significantly decreased (corrected *P* = 0.032) (Fig. 4F). At the uncorrected threshold, interhemispheric FC within the parietal lobe was increased (*P* = 0.025), whereas interhemispheric FC among subcortical structures (*P* = 0.026) and interhemispheric FC between subcortical structures and the frontal lobe (*P* = 0.026) were reduced (Fig. 4F). At the regional level, 2 parietal regions in KO monkeys, including area 7op (corrected *P* = 0.011) and area 5d (corrected *P* = 0.011), showed significantly increased FC density (FCD) (Fig. 4G and H). Additional differences were observed at the uncorrected threshold, including increased FCD in superior parietal lobule area (PEa) (*P* = 0.007) and reduced FCD in 3 frontal regions—area 24a/b (*P* = 0.010), area 14 (*P* = 0.036), and area 10 (*P* = 0.046)—as well as in the accumbens (Acb; *P* = 0.008) and putamen (Pu; *P* = 0.017) (Fig. 4G and H).

Beyond FC, we further characterized differences in brain network topology between WT and KO monkeys. The KO monkeys presented a significant reduction in the global small-world parameter *σ* (Fig. 4I, corrected *P* = 0.007), reflecting altered balance between local clustering and long-range connectivity within the analyzed network. Degree centrality (Dc) analyses revealed significant increases in 3 parietal regions, including PEa, area 5d, and area 7op, as well as in one frontal region, area 8A, whereas the putamen showed a significant decrease in Dc (all corrected *P* < 0.05; Fig. S6A to C). Additional Dc changes were observed at the uncorrected threshold in frontal regions, parietal regions, and subcortical structures, including increased Dc in presupplementary motor area (preSMA), dorsal premotor cortex, and areas 1-2, and reduced Dc in area 24a/b, area 3a/b, area 10, area 9, area 46d, area 13, area 24c, and accumbens (Fig. S6A to C). Furthermore, nodal clustering coefficient (NCp) also showed significant group differences (corrected *P* < 0.05) in multiple regions (Fig. S6D to F). Specifically, NCp was significantly increased in several frontal and parietal regions, including SMA, area 3a/b, area 24c prime, and preSMA, whereas area 25 and area 24a/b showed significantly reduced NCp. Additional NCp differences at the uncorrected threshold were observed in frontal, parietal, and subcortical regions, including increased NCp in secondary somatosensory cortex, ventral premotor cortex, hypothalamus, areas 1 and 2, M1, lateral intraparietal area, and area 8B (Fig. S6D). Detailed regional results are provided in Tables S4 to S6.

To assess the behavioral relevance of the regional neuroimaging abnormalities, we performed exploratory brain–behavior correlation analyses using 3 representative behavioral measures and neuroimaging measures that showed significant group differences after FDR correction. Partial Spearman correlations controlling for species and genotype showed that working memory accuracy at *B* = 2 s was positively associated with cortical thickness in area 24a/b prime (*P* = 0.015) and with Dc of the putamen (*P* < 0.001; Fig. S7). No significant associations were found between neuroimaging measures and RL perseverative errors or cobra-minus-neutral retrieval latency. Together, these results indicate that regionally selective cortical structural and subcortical network abnormalities may be associated with the working memory impairment observed in giant ANK2 KO monkeys.

### Physical and hematological parameters in giant *ANK2* KO monkeys

There were no significant differences in physical development (Fig. S8) and echocardiogram indices between the giant *ANK2* KO and WT monkeys, consistent with our previous report [11], except for a lower aortic valve velocity in KO monkeys (Table S7). These observations suggested that KO of giant *ANK2* may not cause problems with the heart and other organs although previous studies have shown an association of *ANK2* mutations with cardiovascular diseases and other diseases [15–17,36].

**Fig. 5.**
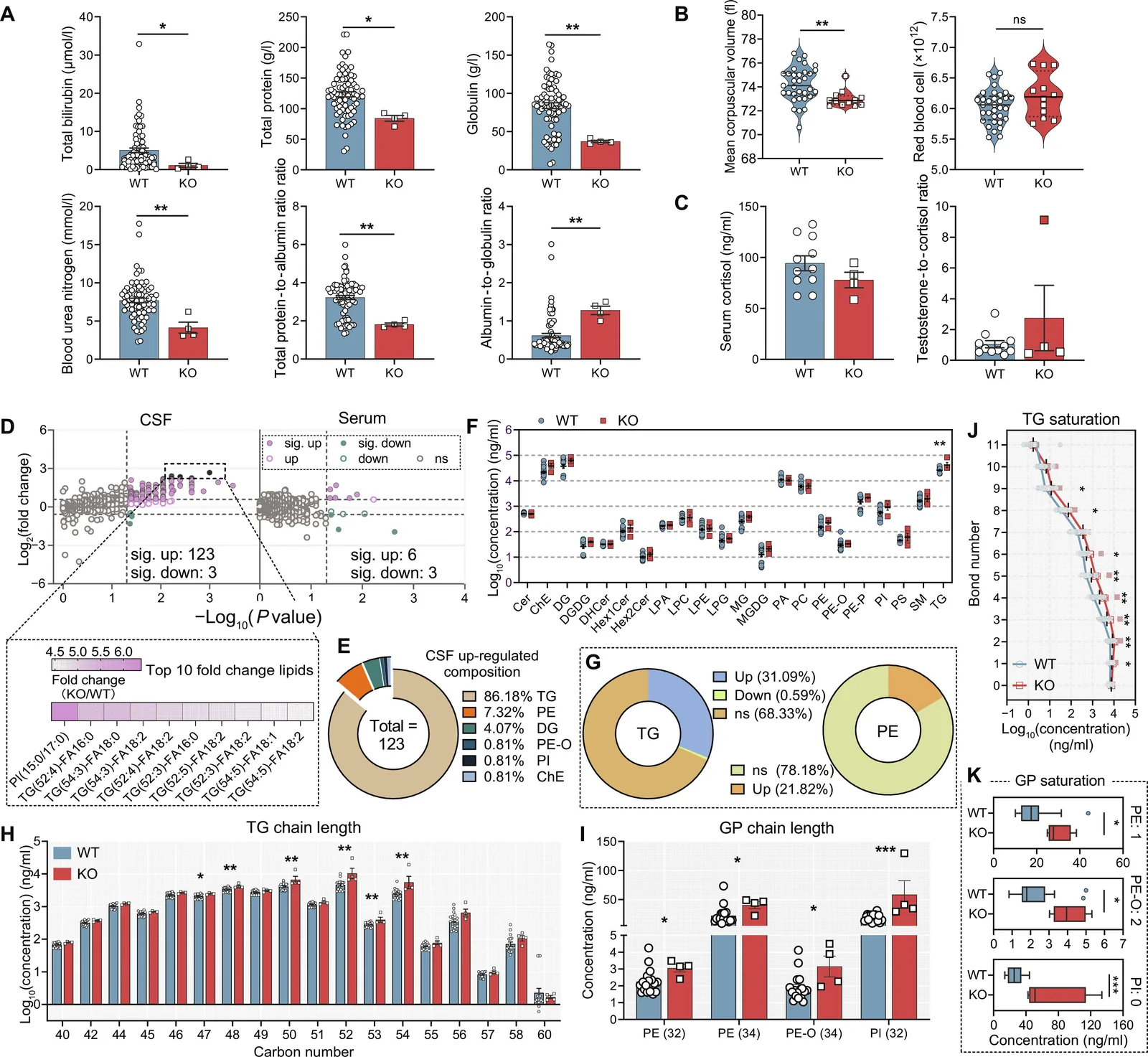
Altered hematological parameters and lipidomic species in cerebrospinal fluid (CSF) in the giant *ANK2* knockout (KO) and wild-type (WT) monkeys. (A) Comparison of the blood biochemical parameters between KO (*n* = 4, including 2 rhesus and 2 cynomolgus monkeys) and age-matched WT monkeys from both species (*n* = 78, including 58 rhesus and 20 cynomolgus monkeys). (B) Comparisons of mean corpuscular volume and red blood cell between KO (*n* = 4) and WT (*n* = 11, including 5 rhesus and 6 cynomolgus monkeys) groups. The data denoted a single measure for an individual, total 3 measurements during the development. (C) Comparison of cortisol and testosterone-to-cortisol ratio between KO (*n* = 4) and WT (*n* = 11) monkeys. The KO monkey (T105) showing an outlier value was marked in red. (D) Changes in lipid species in CSF and serum between KO (*n* = 4) and age-matched WT (*n* = 17, including 11 rhesus and 6 cynomolgus monkeys) groups. The top 10 fold change species (including 1 phosphatidylinositol [PI] and 9 triglycerides [TG]) were denoted as black points and were zoomed in the black dashed box. At the uncorrected threshold, significant up-regulation (sig. up) and down-regulation (sig. down) were defined by pink and green dot respectively, while up-regulation (up), down-regulation (down), no significance (ns) were defined by pink, green, and black circle respectively. (E) Composition of increased lipid species in CSF between WT and KO groups, showing over 85% of lipid species were TG. (F) Concentration of each lipid class between KO and WT monkeys. (G) Percentage of changed species in TG class and phosphatidylethanolamine (PE) class between KO and WT monkeys. (H) Comparison of concentrations of TGs with different carbon chain lengths between KO and WT monkeys. (I) Comparison of concentrations of lipid species in glycerophospholipids (GP) with different chain lengths. Higher concentrations of PE with 32 and 34 carbons, alkyl-PE (PE-O) with 34 carbons, and PI with 32 carbons were observed in KO monkeys compared to WT monkeys. The carbons numbers were in parentheses. (J) Increased unsaturated TG in KO monkeys compared to WT monkeys. The *y* axis presented the number of carbon–carbon double bond, with 0 referring to saturated TG and 1 to 11 representing unsaturated TGs. (K) Box plot showing the saturation status of GP. The PE with 1 double bond (PE: 1), PE-O with 2 double bonds (PE-O: 2) and saturated PI with no double bond (PI: 0) were increased in KO monkeys compared to WT monkeys. ns, no significant; *P < 0.05, **P < 0.01, and ***P < 0.001, KO versus WT, at the uncorrected threshold; Student’s *t* test or Mann–Whitney *U* test. In the violin plot in (B) and box plot in (K), the center line represents the median, the box indicates the interquartile range, and the whiskers extend to 1.5 times the interquartile range to illustrate the full data distribution. Other data were presented as means ± SEM.

In the blood biochemistry test, we found significantly lower levels of total bilirubin (*P* = 0.037), total protein (*P* = 0.037), globulin (*P* = 0.007), blood urea nitrogen (*P* = 0.007), total protein-to-albumin ratio (*P* = 0.003), higher alanine aminotransferase (*P* = 0.022), and albumin-to-globulin ratio (*P* = 0.003) in the KO group (*n* = 4) compared to the age-matched WT group with large sample size (*n* = 78) (Fig. 5A). Moreover, the KO monkey exhibited a significantly lower mean corpuscular volume (*P* = 0.002) and a marginal significance of a higher red blood cell count (*P* = 0.080) when compared to the WT group (*n* = 11) across 3 measures during the development (Fig. 5B). Other hematological parameters with no significant difference were shown in Table S8. The development-related hormone, i.e., cortisol and testosterone, had no significant differences between the 2 groups (Fig. 5C). However, we found that monkey T105 had abnormally elevated testosterone (5.35 ng/ml) and a high testosterone-to-cortisol ratio (9.12) (Fig. 5C), both exceeded the normal range observed in age- and species-matched WT males (testosterone-to-cortisol ratio =1.22 ± 0.20, *n* = 8, means ± SEM). At age 3, it was also larger (body length, 53 cm) and heavier (body weight, 6.08 kg) than male control monkeys (length, 44 ± 0.44 cm; weight, 3.7 ± 0.04 kg; *n* = 3, means ± SEM). We assumed that these changes may be associated with the abnormal behavior of this monkey (Fig. S2G).

### Up-regulated triglyceride and glycerophospholipids in cerebrospinal fluid in giant *ANK2* KO monkeys

The lipid metabolism was closely related to brain development, aging, and disorders [37,38]. We therefore performed a targeted lipidomic profiling of cerebrospinal fluid (CSF) and serum using liquid chromatography–mass spectrometry (Fig. S9A). At the uncorrected threshold, whereas few lipid molecules in serum were altered in KO versus WT groups, CSF showed extensive changes (fold change > 1.5 or < 0.67, *P* < 0.05), prompting our subsequent focus on CSF (Fig. 5D and Table S9). Notably, almost all differential lipids were up-regulated. While a phosphatidylinositol (PI) species showed the highest fold change (Fig. 5D), the vast majority fell under that of glycerolipids where triglyceride (TG) species predominated, and the second most highly changed category were glycerophospholipid (GP) that was mainly composed of phosphatidylethanolamine (PE) (Fig. 5E). Consistently, overall TG concentration was higher in KO monkeys (*P* = 0.004, Fig. 5F) than in WT monkeys, while total glycerolipids and all other lipid categories did not change (Fig. S9B). Note that up-regulation affected a sizable fraction of both TG and PE species (Fig. 5G).

We next examined lipid structural changes, i.e., chain length as carbon number and saturation as double-bond count, where a higher double-bond indicated greater unsaturation. At the uncorrected threshold, KO monkeys showed elevated TG concentrations across most chain lengths, with increases in mid-length species (Fig. 5H, all *P* < 0.05). Moreover, GP species including PE, PI, and alkyl-PE (PE-O) at specific chain lengths were also increased in KO monkeys (Fig. 5I, all *P* < 0.05). Unsaturated TG concentrations were preferentially elevated in moderately unsaturated species, raising the total concentration of unsaturated TG and the unsaturated TG fraction in KO monkeys (Fig. 5J and Fig. S9C, all *P* < 0.05). The membrane fluidity was assessed on the basis of GP-associated indicators, i.e., saturated fractions and unsaturated concentrations of GP, phosphatidylcholine (PC), and PE class (including PE, PE-O, and alkenyl-PE [PE-P]) following a previous study [38]. The saturated fractions were negatively correlated with fluidity, while unsaturated concentrations were positively correlated. However, no significant changes in fluidity were detected between the 2 groups (Fig. S9D). Despite this, specific PE/PE-O species with fewer double bonds and saturated PI were significantly increased in KO monkeys (Fig. 5K, all *P* < 0.05). Collectively, our CSF lipidomic analyses showed that giant *ANK2* KO up-regulates TG and GP species in monkey CSF, potentially linking KO of giant *ANK2* to the disruption of brain lipid homeostasis in monkeys.

### Dysregulated transcriptomic immune profiling in peripheral blood in giant *ANK2* KO monkeys

**Fig. 6.**
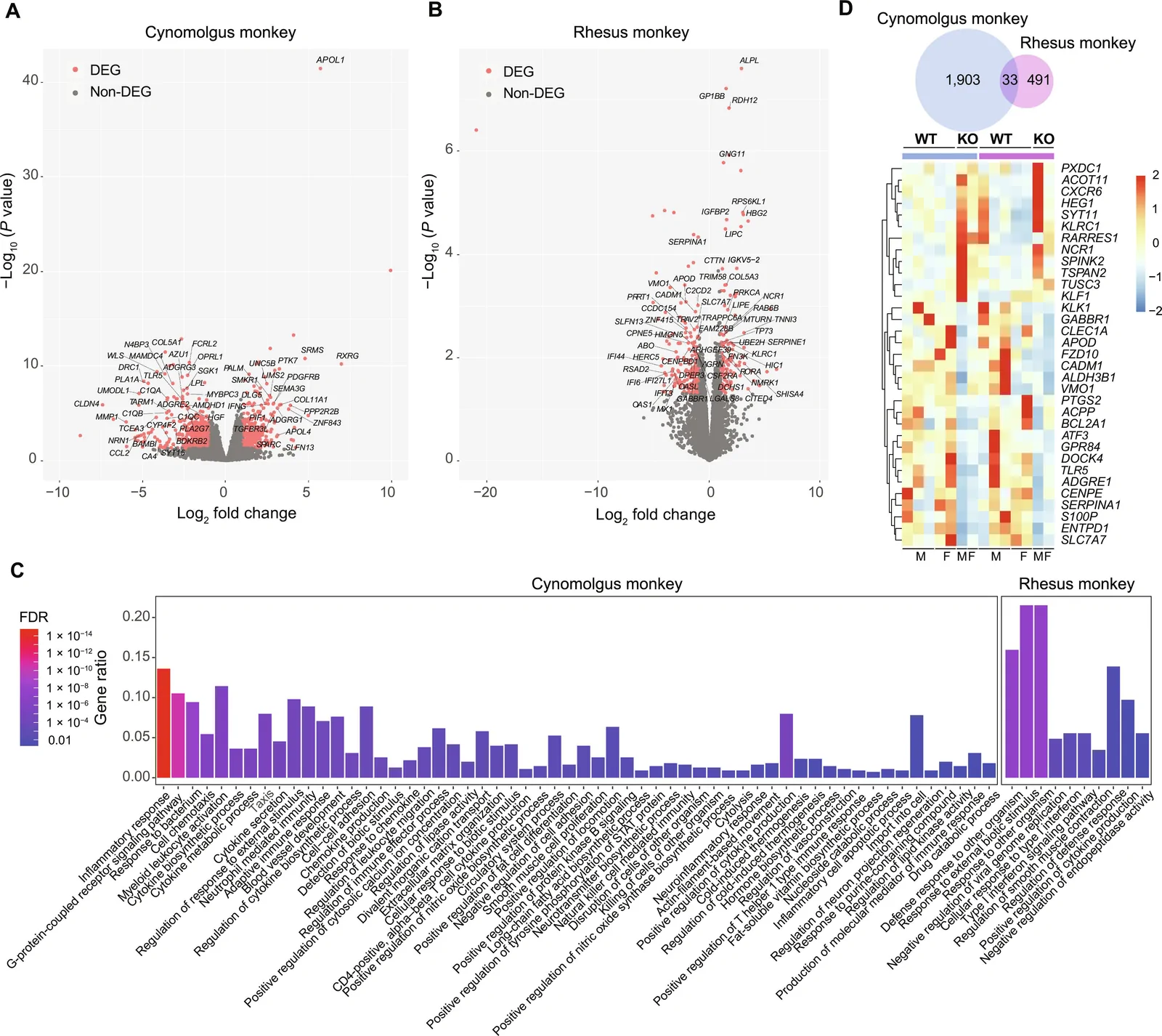
Transcriptome analysis revealed dysregulated immune process in the giant *ANK2*-KO (KO) monkeys. (A and B) Top differentially expressed genes (DEGs) in KO monkeys compared to wild-type (WT) monkeys. (C) Enriched biological pathways of DEGs between KO and WT monkeys. Gene ontology, biological process, FDR < 0.05. (D) Heatmap of 33 shared DEGs between cynomolgus and rhesus monkeys. The DEGs between KO and WT groups were defined by |logFC| > 1 and *P* < 0.05. Rhesus group, 2 KO and 5 WT monkeys; cynomolgus group, 2 KO and 5 WT monkeys. M, male; F, female; STAT, signal transducers and activators of transcription.

To further investigate the physiological impact of giant ANK2 ablation, we compared peripheral blood transcriptomic profiles between KO and WT monkeys. Among genes with over 2-fold expressional changes in KO cynomolgus monkeys (1936 genes) and KO rhesus monkeys (524 genes) (|log_2_ fold change| >1 and *P* < 0.05), a number of genes related to immune processes were dysregulated at the uncorrected threshold. Gene expression and differential expression patterns (represented by log_2_ fold change) were strongly correlated between the 2 monkey species (Fig. S10). The observed differences in the number of differentially expressed genes (DEGs) between species might reflect underlying genomic variability [39] or differential compensatory responses to *ANK2* depletion. Genes with important roles in inflammatory regulation, including *TLR5*, *CCL2*, *AUZ*, *TARM1*, and *CLEC4D*, were down-regulated in KO cynomolgus monkeys (Fig. 6A). Key molecules involved in the type I interferon signaling pathway, such as *IFI6*, *IFIT3*, *OAS1*, *OASL*, *RSAD2*, and *MX1* were also remarkably down-regulated in KO rhesus monkeys (Fig. 6B). DEGs in these 2 groups of KO monkeys were enriched in immune-process-related pathways, especially for pathways related to cytokine production (GO:0001819, positive regulation of cytokine production; Fig. 6C). Among commonly dysregulated genes shared by both KO monkey species, a large proportion of genes were related to the immune process, including *TLR5*, *CLEC1A*, *ATF3*, *GPR84*, *ENTPD1*, *ADGRE1*, *KLRC1*, *CXCR6*, and *NCR1* (Fig. 6D). These results were consistent with immune system abnormalities and cytokine alterations in patients with ASD reported by previous studies [40]. Furthermore, the mRNA expression levels of immune-related genes and the protein levels of cytokines and other immune-related proteins were significantly altered in the peripheral blood of patients with ASD [40,41]. Future validation experiments should be performed to determine whether these DEGs and enriched pathways participate in brain pathology caused by the giant *ANK2* KO.

## Discussion

The *ANK2* gene is highly conserved, with its protein AnkB expressed in organs, such as the brain, heart, skeletal muscle, and thymus [16], and its mutations associated with many diseases, especially NDDs, including ASD [17,36,42]. In our previous study, we generated rhesus and cynomolgus monkeys deficient of the giant *ANK2* isoform AnkB-440 [11]. These KO monkeys from both species exhibited massive brain volume loss at early ages (6 months, 1 year, and 2 years) [11] and sustained this loss up to 4 years of age or older. We proposed that this structural defect would cause neural impairments that emerge with development and aging, rather than in infancy [11]. In this study, longitudinal studies of the giant *ANK2* KO monkeys revealed hyperactivity, cognitive deficits (in working memory, flexibility, and OL), and disrupted social interactions, aligning with human *ANK2* mutation phenotypes [43]. However, the hyperactivity contrasted with the mice carrying an analogous AnkB-440 mutation, which showed reduced activity [10], suggesting species-specific differences. In addition, the giant *ANK2* KO monkeys displayed emotional dysregulation, and adolescent KO monkeys exhibited social deficits and a disrupted social hierarchy or affiliation in the colony, probably due to increased avoidance rather than approaching behavior, aligning with prenatally valproate-induced ASD-like rats and schizophrenia [44,45]. Although these KO monkeys displayed social deficits, they did not exhibit overt stereotypical behaviors; thus, we cannot conclude that they fully recapitulate ASD. Nevertheless, they may model specific symptomatic features of NDDs. Of note, these behavioral abnormalities were not present during infancy or juvenility [11] but emerged in KO monkeys as social hierarchy structures matured. Consequently, detecting social behavioral differences in infant monkeys is inherently challenging.

Giant *ANK2* KO monkeys showed persistent cerebral ventricular enlargement, resulting in morphological alterations in the occipital, temporal, and posterior parietal lobes, as well as some subcortical regions. Outside these regions of structural change, they exhibited reduced GMT in several frontal cortical regions, particularly within cingulate areas, which may be associated with impairments in working memory and cognitive flexibility found in this study. Notably, difficulty in learning an operant task mirrored intellectual disabilities observed in *ANK2* mutation patients [43]. Moreover, rs-fMRI revealed significantly increased FCD in parietal areas, with additional reductions in frontal and subcortical regions observed at the uncorrected threshold, indicating altered regional importance within the analyzed network. Network integration and segregation were also disrupted, characterized by a significantly reduced small-world coefficient and significant region-specific changes in NCp, with additional NCp differences observed at the uncorrected threshold, which may contribute to the observed behavioral abnormalities [46,47]. Importantly, the regional neuroimaging abnormalities showed behavioral relevance: Poorer working memory performance was associated with reduced cortical thickness in area 24a/b prime and lower Dc of the putamen. These findings provide evidence linking regionally selective brain abnormalities to the cognitive phenotype of giant *ANK2* depletion, although the exploratory nature and small sample size of the correlation analyses warrant validation in larger cohorts.

Intriguingly, in mice, homozygous deletion of *ANK2* restricted to cortical and hippocampal excitatory neurons results in ASD-like behaviors and leads to juvenile seizure-related mortality [48]. In human patients, *ANK2* mutations have been associated with epilepsy [43,49]. Moreover, the *ANK2* variants have been linked to an inherited disorder known as “AnkB syndrome”, characterized by a range of cardiac arrhythmias and cardiomyopathy [15,16]. We observed no epilepsy or cardiac dysfunction in the giant *ANK2* KO monkeys during the continual investigation up to 7 years of age and no elevated neuronal synchronies as seen in epileptic mice [8]. However, the disrupted small-world coefficient in KO monkeys aligned with the findings in childhood absence epilepsy syndrome [50]. Long-term monitoring of cardiac function in these KO monkeys is warranted, particularly at key developmental stages such as middle and old ages. In addition, the KO monkeys with cerebral ventricular enlargement, a feature previously reported in patients with schizophrenia [51], exhibited significantly higher movement speed. This was compatible with the positive symptoms observed in schizophrenia [52]. Future studies could investigate whether these monkeys also display impaired sensory gating and auditory deficits, a well-established clinical hallmark of the psychiatric disorder, to confirm whether these KO monkeys had schizophrenia-like symptoms.

Consistent with our previous study [11], most physical indices showed no significant differences between KO and WT monkeys. Although there were no significant intergroup differences in body weight across development, 2 outlier individuals deserve attention. One male KO rhesus monkey (T105) exhibited social isolation, necessitating separate housing for its welfare. It showed abnormally elevated testosterone, which is related to aggression and may contribute to ASD [53], and a larger body size relative to age- and species-matched WT monkeys. This particular case of excessive development might represent an alternate NDD manifestation, suggesting a link between abnormal hormone profiles and alterations in behavior and physical growth [54]. However, it remains unclear whether the behavioral alterations in this monkey are associated with such hormonal abnormalities or the gene deletion. Probably, these 2 factors may interact with each other, and further experiments are required to verify their causal relationship. On the contrary, one dwarf female cynomolgus KO monkey (T88) exhibited consistently low body weight (below 2.75 kg) from infancy through adulthood and reduced blood glucose levels (fasting, 2.4 mmol/l; 1 h after feeding, 2.6 mmol/l). Given that the absence of CRISPR/Cas9 off-target effects has been confirmed in our previous study [11], this observation suggested that giant *ANK2* KO may cause complex, variable physiological outcomes, potentially influenced by KO efficiency, gender-dependent response to gene editing, and species-specific factors, although a small fraction of genes (3.7%) showed differential expression between the 2 species [39].

Compared with age-matched WT monkeys, the giant *ANK2* KO monkeys exhibited several alternations in hematological and biochemical parameters, suggesting that the giant *ANK2* KO may exert broad effects on monkey’s physiological processes. Furthermore, our data showed that *ANK2* KO monkeys have elevated CSF levels of TG and GP compared to controls, while blood lipid levels remain unchanged. These KO animals also exhibited cognitive deficits. Although regional neuroimaging abnormalities correlated with behavioral changes, the co-occurrence of CSF lipid alterations and cognitive impairment was well documented. This association was supported by previous findings in both humans and rodents [55,56] and was corroborated by clinical evidence identifying TG as strongly associated with cognitive decline in Alzheimer’s disease [57] and in aging individuals with cognitive dysfunction [58]. Furthermore, the concurrent increase in diacylglycerols and PI (15:0/17:0) may point to aberrant PI signaling in the brain following giant *ANK2* deletion, a pathway recently implicated in neurodevelopment [59]. However, the current data are limited at the uncorrected threshold due to the small sample size and do not establish a causal link nor elucidate the underlying mechanisms. Whether and how these lipid changes contribute to the cognitive deficits remain open questions that necessitate further investigation. For example, it would be valuable to determine whether the elevated TG/phosphatidylglycerol species are localized to specific cognition-related brain regions and to explore their potential connections to excitatory/inhibitory transmission via pyramidal cells and interneurons, once brain tissues or organoids become available.

Peripheral blood transcriptome analysis suggested dysregulated immune-related genes (e.g., *TLR5*, *CCL2*, *AUZ*, *TARM1*, and *CLEC4D*) and associated pathways in giant *ANK2* KO monkeys. Whether and how these molecules contribute to the pathological mechanisms underlying NDD- or ASD-like behaviors associated with ANK2 mutations await further studies using in vivo targeted gene editing or pharmacological manipulation. One interesting question arising from these data is why more DEGs were observed in cynomolgus monkeys compared to rhesus monkeys following giant ANK2 KO. Focused study with large cohorts of KO animals would help clarify this species-specific difference.

This study has several limitations. First, the small cohort of giant *ANK2*-deficient monkeys necessitates validation with a larger sample. We are currently expanding the breeding of these animals to reduce variability and obtain more robust insights into the functional alterations resulting from giant *ANK2* KO. Second, our MRI and rs-fMRI analyses were necessarily confined to anatomically reliable frontal, anterior parietal, and selected subcortical regions. The observed structural, FC, and graph theoretical alterations characterize abnormalities within this retained frontoparietal–subcortical network, while their generalizability to the excluded regions and whole-brain network organization remains to be established. The lack of brain tissues at different developmental stages prevented molecular/cellular analyses from examining neuronal changes. The expanded cohort and establishment of brain organoids combining with emerging technologies on single-cell sequencing, such as artificial-intelligence-based spatial cellular profiling and single-cell-specific causal network [60,61], may provide more insights into dynamical processes of brain loss and its underlying mechanism. Third, although lipidomic and transcriptomic analyses suggested alterations in metabolic and immune pathways associated with *ANK2* depletion, these data remain to be further validated, and further functional validations are necessary to elucidate the molecular mechanisms underlying these observed changes.

In summary, we conducted a longitudinal study of monkeys deficient in giant *ANK2*, establishing a comprehensive lifespan timeline of behavioral, neuroimaging, and physiological changes in these KO monkeys. Extending our earlier observations from infancy [11], these KO monkeys developed progressive behavioral abnormalities with age, including cognitive, emotional, and social deficits. They also exhibited alternations in brain structure and function, alongside dysregulated lipidomic and immune-related transcriptomic profiles. These findings suggest that giant *ANK2* depletion in NHPs recapitulates key phenotypic features of human NDDs, offering cross-species insights into brain functional development and distinct dimensions of disease pathogenesis, and can thereby aid future mechanistic and therapeutic research.

## Materials and Methods

For details about the materials and methods, please see the Supplementary Materials.

## Data Availability

The data are freely available upon request.

## Supplementary Materials

Supplementary 1 Materials and Methods Figs. S1 to S10 Tables S1 to S9 References [62–85]
