# Supplementary material for: Longitudinal Characterization of Giant ANK2-Depleted Monkeys Suggests Neurodevelopmental-Disorder-Like Phenotypes
Source: Research (Wash D C). 2026 Sep 7;9:1424. doi: 10.34133/research.1424 (PMC13547740; doi:10.34133/research.1424)
Supplement: Supplementary file 1 [file research.1424.f1.docx]

**Online Supplementary Materials**

**Longitudinal characterization of giant *ANK2*-depleted monkeys suggests neurodevelopmental disorder-like phenotypes**

**Hong-Di Huang et al.**

**Corresponding author: Yong-Gang Yao et al.**

The file includes:

Supplementary Materials and Methods

Supplementary Fig. S1 to S10

Supplementary Tables S1 to S9

**Materials and Methods**

**Animals**

A total of four monkeys with giant *ANK2* knockout (KO) (two *Macaca mulatta* (T105, male; T111, female) and two *Macaca fascicularis* (T87, male; T88, female), of which the detailed construction methodology and validation data were first reported in our previous study [11], were used in current study. Eleven age-, gender-, species -matched wild-type (WT) healthy controls (five *Macaca mulatta*, two females and three males; six *Macaca fascicularis*, three females and three males) were mainly included, for social interaction test, more wild-type monkeys were used for controls. For the hematological and lipidomics study, a large sample size of age-, gender-, species(part)-matched WT individuals were used in the same batch of measurement, and the related sample information was listed in the respective figure legends. All animals were housed under standardized conditions with 60% humidity, a temperature of 21±5 °C, and a 12-h light/dark cycle (light on at 7:00 AM). The monkeys received chow, fresh fruits, vegetables, and water *ad libitum*, with daily veterinary care. All procedures complied with the guidelines approved by the Institutional Animal Care and Use Committee (IACUC 20004) of the Kunming Institute of Zoology (KIZ), Chinese Academy of Sciences (CAS), with efforts made to minimize suffering and the number of animals.

**Experimental design**

We conducted a longitudinal measurement of behavior, neuroimaging, physiology, lipidomics, and peripheral blood RNAseq in the giant *ANK2* KO monkeys, and age-, gender, species (part)-matched WT monkeys (Fig. S1). KO and WT animals of the same species were compared unless otherwise stated. A series of behavioral tests was administered at appropriate ages as follows: The spatial delayed response (SDR) and visual discrimination/reversal learning (VD/RL) tasks [35, 62] were conducted when the monkeys were between 2.3 and 3.4 years old. The emotional response (ER) test [63] was performed at 3.5 years of age. Social hierarchy and interaction [35, 64] were assessed at 5.5 years of age, after the monkeys had been housed in three separate colonies without intervention for one year. Deep learning-based behavioral analysis, home-cage activity and sleep, manual dexterity, neurological function, exploratory behavior tests, the operant learning (OL) task and three-chamber social interaction test (SIT) were performed between 6 and 7 years of age, following the methods described in our recent studies [34, 35, 65-67]. The monkeys had a magnetic resonance imaging (MRI) test at an age of 4.2 years, and periodically physical and physiological, lipidomics and peripheral blood RNAseq measurements were taken from 1 to 7 years of age.

**Behavioral tests**

To minimize training variability across individuals, all behavioral testing was conducted using a unified, criterion-based training framework. Monkeys in both groups were trained under identical training schedules, including a fixed number of trials per session, and were required to reach predefined performance criteria before proceeding to the subsequent stages or formal testing. All tasks were performed by the same experienced experimenter.

***Measurement of neurological function.*** The neurological function was scored using a behavioral scale developed in our previous reports [66, 67]. The total score of the scale was 50 points, with 16 points assigned to the motor system, 9 points to skeletal muscle coordination, and 25 points to the sensory system. A score of 0 corresponded to normal behaviors, whereas higher scores represented severe neurological dysfunction. Two well-trained observers conducted the scoring. Monkeys from both species (rhesus and cynomolgus) were measured.

***Measurement of home-cage activity and sleep.*** To evaluate locomotive activity in the two monkey species, an actigraphy device (ActiGraph GT9X Link, ActiGraph LLC, USA) [34, 35] was mounted on each animal’s collar. Home-cage activity and sleep were monitored continuously over 11 days, with the first day excluded for acclimation and the subsequent nine days used for analysis. The sleep period was defined as 19:00 to 7:00. Data were sampled at 30 Hz with 60-second epochs [35] and processed using ActiLife 6 software (ActiGraph LLC) to convert raw triaxial acceleration and rotation into analyzable metrics. Average vector magnitude counts (VMC) quantifying the daily activity and sleep parameters (including total sleep time and sleep efficiency) were measured and processed as previously described [35]. Monkeys from both species were measured.

***Exploratory behavior test.*** A laser-pursuit test was employed to assess monkey exploration behavior [35]. Briefly, a familiar experimenter stood 1 meter away from the home cage and presented a neutral icon to the monkey using three different patterns: rotating the icon clockwise around the cage, rotating it counterclockwise (each with a 5-second cycle), and shaking it near the monkey (within a 5-10 cm range). The duration and frequency of the monkey’s responses including mouth licking, hand touching, and visually tracking the icon with head/body movement, were recorded and analyzed. Monkeys from both species were tested.

***Measurement of manual dexterity.*** A vertical slot task which required the animals to retrieve a 7×7×13mm hawthorn strip inside the slot was conducted to measure the manual dexterity of monkeys’ preferred hands [65, 66]. The task was performed 20 trials per session and each monkey was tested in 5 sessions. A camera (HDR-CS405, Sony, Japan) recorded the entire procedure for subsequent frame-by-frame analysis using Observer software (Noldus Information Technology B.V., Netherlands). Before collecting testing data, monkeys were trained to complete 20 trials within 20 minutes for three days, so as to adapt for a new task. The dexterity was scored based on the average duration of each trial to successfully retrieve, the percentage of successful retrievals, and the percentage of precision grasp (opposition of the thumb and the index finger). Monkeys from both species were tested.

***Decomposition of free-moving kinematics and locomotion.*** The 3D-motion learning toolkit consisted of a multi-view capture system and a machine learning assisted computational framework, which enabled precise unmarkable tracking and quantitative analysis of animal’s movements [68], as demonstrated in our recent studies [34, 35]. Briefly, the multi-view system recorded monkey’s locomotive behaviors using four RealSense D435 cameras (Intel, USA) positioned 1.1 m from a three-sided transparent 100 cm³ testing cage, ensuring full coverage. Cameras sampled 30 fps footage (848×480 pixels) after the monkey was transferred into the cage. The computational framework was integrated into the BehaviorAtlas NHP software (v1.01, Guangdong BayONE Scientific Co., Ltd., China). A well-trained model was generated by labeling 21 key body parts on the monkeys from extracted 4200 extracted frames based on DeepLabCut methodology (BehaviorAtlas Pose Labeler V1.0.0, Guangdong BayONE Scientific Co., Ltd., China). The model automatically recognized the monkey’s 21 body parts in targeted footage and reconstructed the 3D skeleton information of the monkey, from which the 46 kinematic parameters (Table S1) were subsequently extracted. With this information, 14 types of locomotive behaviors were decomposed by supervised classification. The behaviors (Table S2) were categorized as with/without apparent displacement (AD/NAD). Principal component analysis (PCA) was performed on the standardized kinematic parameters. To validate the accuracy of 2D coordinate recognition for individual monkeys, we randomly selected 1,000 images and achieved accuracy rate exceeding 90% across all groups. For three‑dimensional trajectories reconstruction, we plotted the 3D coordinates of 21 body landmarks. To assess the performance of spontaneous behavior recognition, we randomly sampled 30 frames from each of 50 datasets, which yielded a recognition success rate of 87.44%. We only tested for the rhesus monkeys (KO, *n* = 2; WT, *n* = 5) because the deep learning-based models were optimized for rhesus monkeys, but not for cynomolgus monkeys. Each monkey underwent two 35-min recording trials with an intertrial interval of a couple of days. Every trial was used as a sample for statistical analysis owing to the limitation of sample size.

***Spatial delayed response (SDR) task*.** The SDR task was performed by using the Wisconsin General Test Apparatus (WGTA) [35]. We grouped all rhesus and cynomolgus monkeys into two groups (KO group, *n* = 4, including two rhesus KO and two cynomolgus KO monkeys; WT group, *n* = 9, including three rhesus and six cynomolgus monkeys, with the exclusion of two rhesus monkeys who refused to perform the task) to maximize the sample size for each group. At first, monkey underwent a period of habituating WGTA and experimenter. The monkey was then trained with *B* = 0 s to learn the procedure, until achieving a criterion (86.7%, 26 correct choices out of 30 trials for consecutive 3 sessions). Subsequently, the assessment began at *B* = 2 s. Then, the B delay increased by 3 s if the monkey achieved the predefined performance threshold, otherwise, the B delay would decrease by 1 s. The maximum of 10 training sessions were allowed if the monkeys failed to meet the performance criteria. A total of 30 trials per daily session were conducted. The rewards were pseudo-randomly placed in the left or right well across a session. Five delay lengths (*A–E*) were semi-randomly assigned per session: *A* = *B*×0 s (second), *B* = *B*×1 s, *C* = *B*×2 s, *D* = *B*×3 s, *E* = *B*×4 s. The criterion was set as animals would achieve 86.7% (26/30 trials) of correctness for consecutive 3 sessions, in a maximum of 10 training sessions. At first, the monkey was trained with *B* = 0 s to learn the procedure until it achieved the above criterion. Subsequently, the assessment began at *B* = 2 s. Then, the *B* delay increased by 3 s if the monkey achieved the criterion, otherwise, the *B* delay would decrease by 1 s. The individual monkey may perform a different maximum B delay. Two WT monkeys were unable to complete the SDR tasks due to their timid personalities. The mean percentages of correct choice at *B* = 2 s, maximum B delay for each monkey, and mean number of corrections at each delay across all trials were calculated to indicate the ability of the monkey’s working memory.

***Visual discrimination and reversal learning (VD/RL) task*.** The WGTA-based VD/RL task, which assessed the association learning and cognitive flexibility in NHPs, was conducted following the SDR task [62]. Prior to the VD/RL task, all monkeys underwent an adaptation phase during which they were trained to retrieve rewards from wells covered with two identical cardboard pieces. Each animal was required to complete 40 trials per session per day and to achieve a performance criterion of 87.5% correct responses. In the VD stage, the animal was presented with two different, novel cards and allowed to choose either of them. One was covering a food reward (S+), whereas the other was non-rewarded (S-). By training for 40 trials per daily session, the monkey learned which card was associated with the reward and chose the S+ card to retrieve it, indicating that it was able to distinguish between the two different cards. After the monkey achieved a criterion of 87.5% (35/40 trials) for 3 consecutive sessions, the RL stage commenced by reversing the association between the cards and the reward. The task ended after the monkey achieved an 87.5% correct rate or a maximum of 20 sessions. Percentages of correct choices (chose S+) of each session, trials to criterion, errors before achieving criterion and errors before consecutively correcting for three trials after reversal were calculated.

***Operant learning (OL) task*.** The monkey was trained to receive a food reward by pressing a lever on a customized operant apparatus (Anilab, Anlai Scientific Instrument (Ningbo) Co., Ltd., China). A successful lever-press triggered the release of a fruit-flavored pill (0.25 g) into a receptacle located 140 cm from the lever. The monkey was initially guided by the experimenter to press the lever for a maximum of 0-30 trials (contingent on individual monkey’s learning ability) and then allowed to freely engage with the lever for a one-hour session, with a total of 3 to 6 sessions daily, and a maximum of 50 presses per session. This training course ended after the monkey successfully performed 50 autonomous presses per session for three consecutive sessions. The number of manual training trials before the monkey ended the guidance was recorded. The monkeys were food-restricted before training and provided food to normal nutritional intake after daily training, and monkeys from both species were trained.

***Emotional responsiveness (ER) test*.** We used the procedure adapted from Pujara et al. [63]. Monkeys were pretrained with a 30-trial session per day, in which four neutral “junk” objects were randomly presented one at a time for 3 trials, interleaved with 18 empty-box trials. Training continued until the animal retrieved the reward within ~5 seconds on each trial, ensuring reliable retrieval before proceeding to the later test. Food preference was evaluated by randomly placing two of four reward types (hawthorn candy, peanut, raisin, sweet potato) on the left and right sides of a transparent box (55×20×15 cm) in the WGTA. A total of six pairs of rewards were presented 6 times across 36 trials/session. The monkey’s preferred food was identified as the most frequently chosen across 5 sessions. The emotional response (ER) test was then conducted, comprising 5 sessions (each with 10 trials, spaced 2 days apart). Each trial featured a novel neutral object or a negative stimulus (a rubber cobra/spider) placed in the box with a favored reward at the center-top. Stimuli were presented semi-randomly once per session (30s intertrial interval; max 45s exposure). Negative stimuli were excluded from the first 4 trials and consecutive presentations. Food-retrieval latency (from trial start to reward retrieval) and approach/defense behaviors were scored by video analysis. Approach included forward movement, touching, or eating. Defense included freezing ≥ 3s, startle, aversion ≥3s, or withdrawal to the back of the cage. Approaching (APP) and defending (DEF) might coexist in the same trial. We calculated the approach-defense index (ADI) to quantify the behaviors following the formula: ADI = (P_APP_ - P_DEF_) / (P_APP_ + P_DEF_), in which the P_APP_ or P_DEF_ was the proportion of trials presenting approaching or defending, respectively. Monkeys from both species were tested.

***Social interaction*.** After over one year of acclimatization in three social colonies (excluding KO rhesus monkey T105 as noted by the welfare manager), all monkeys were video-recorded for 21 consecutive days. Social interactions between 8:00-12:00 and 13:00-17:00 over eight days were scored by four experts using Observer™ software (Noldus Information Technology B.V., Netherlands). Behaviors were classified as described in our previous study [35], including proactive social (affiliative, grooming), passive social (affiliative, grooming), aggression, or submission. Grooming was defined as initiating and maintaining physical contact ≥3s; affiliative behavior as sitting within arm’s length, playing, or hugging ≥3s; aggression as threatening, biting, rearing back and opening mouth, roaring, or slapping; and submission as lip-smacking, grimacing, or escaping. Proactive and passive behaviors were measured by duration, while aggression and submission were counted by frequency. Total social interaction time excluded aggressive and submissive events. Social ranks were calculated using weighted David’s scores based on aggression or submission outcomes [65], with higher scores indicating higher rank. Monkeys from both species were tested.

***Three-Chamber social interaction test (SIT).*** The three-chamber SIT was adapted from rodent paradigms [69]. The apparatus had four sequentially connected cages (850×850×850 mm each). Left/right cages were separated from the two central interconnected cages (1 and 2) by grid panels, forming three compartments (Fig. 3F). Two test sets in adjacent quiet rooms served different-sex monkeys, each with a front-facing camera (SONY, 30 fps). Three phases were conducted (Fig. 3F) on only rhesus monkeys due to the limitation of the same-species strangers.

*Habituation*: Monkey explored central cages for 45 min/session/day, three sessions. Time in cage 1 versus cage 2 was analyzed. The side with the longer duration was defined as the preferred.

*Social affiliation*: Toy (non-social stimulus) was placed on monkey’s preferred side, one unfamiliar monkey (stranger 1, social stimulus) was opposite. Monkey was allowed to stay in central cage 1 and 2 for 30 min/session/day, three sessions.

*Social memory*: Toy was replaced with a novel unfamiliar monkey (stranger 2). The monkey interacted with stranger 1 and stranger 2 through visible grid panels for 30 min/session/day, three sessions. Unfamiliar stimulus monkeys and test subjects were matched for sex, age and size, and any aggressive individuals were excluded. “Close” was defined as the test monkey’s entry into the half-cage zone adjacent to the stimulus, reflecting approach behavior. “Far” was defined as the remaining half of the stimulus-side cage plus the full extent of the opposite adjacent cage, reflecting avoidance behavior (Fig. 3G).

**MRI acquisition and data processing**

***Data acquisition*.** The two macaque species (*Macaca mulatta* and *Macaca fascicularis*), with both represented in similar numbers in both the KO and WT groups to prevent the results from being dominated by one species. MRI scanning was carried out for KO and WT monkeys at 4.2 years of age, following the procedure described in our previous studies [35, 67]. Prior to scanning, monkeys were premedicated with atropine (0.05 mg/kg, i.m., HeNan RunHong Pharmaceutical Co., Ltd., China) to reduce salivation. After 5 min, anesthesia was induced by hydrochloric acidulated ketamine (Jiangsu Zhongmubeikang Pharmaceutical Company, 10 mg/kg, i.m.) and maintained with propofol (15 mg/kg/h, i.v.) during the scanning. Anesthesia was continuously monitored for the animal’s safety and signal stability.

The MRI and rs-fMRI data were acquired by a 3.0T MRI scanner (uMR790, United Imaging Healthcare Co., Ltd., China) with a 12-channel head coil at the KIZ. The T1-weight (T1w) images were acquired using a 3D fast spoiled gradient echo (gre_fsp) sequence: voxel size = 0.5 mm isotropic, echo time (TE) = 5.6 ms, repetition time (TR) = 13.01 ms, flip angle (FA) = 8^o^. The T2-weight (T2w) images were acquired using an fse_mx sequence: voxel size = 0.5 mm isotropic, TE = 396.48 ms, TR = 3400 ms, FA = 59^o^. The rs-fMRI images were collected using an echo planar imaging (EPI) sequence (voxel size = 1.5 mm isotropic, TE = 29 ms, TR = 1700 ms, FA = 80^o^), with each session comprising 500 EPI volumes. Reverse phase encoding data were also acquired for EPI image correction.

Because marked abnormalities in ventricular morphology were present in some ANK2 KO monkeys, several adjacent cortical and subcortical regions exhibited excessive deformation during anatomical registration and atlas-based parcellation. Consequently, morphometric and regional MRI/fMRI analyses were restricted to regions with reliable anatomical correspondence across all subjects. Regions were excluded when their boundaries could not be reliably delineated, when cortical surface placement or subcortical segmentation was unstable, or when atlas alignment was deemed unreliable. As a result, the analyses were focused on frontal regions (*n* = 29), anterior parietal regions (*n* = 10), and six main subcortical regions (e.g., the amygdala) per hemisphere (Table S3). All inclusion and exclusion decisions were made based on visual quality control of anatomical reconstruction, surface/segmentation accuracy, and registration quality, rather than on statistical outcomes. The complete list of retained and excluded regions is provided in Supplementary Table S3, and the MRI/fMRI analysis mask is illustrated in Figure S6.

***Structural data and myelination process.*** Structural data were processed using Analysis of Functional NeuroImages (AFNI) [70], FMRIB Software Library (FSL) [71], Advanced Normalization Tools (ANTs) [72] and FreeSurfer [73]. The procedure for generating gray matter and white matter surfaces, and extracting gray matter volume, thickness, and surface area for each cortical region, as well as volume for subcortical regions was in line with our previous studies [35, 67].

In detail, for each animal, the T1w image was registered to the NIMH Macaque Template, NMT version 2.0 [74]. Skull stripping masks were generated using an in-house neural-network-based procedure and were used to support subsequent processing. T2w images were co-registered to the corresponding T1w images using a rigid-body transformation. A bias correction procedure was applied to enhance contrast by combining the T1 and T2 images.

After bias correction, both T1w and T2w images were further denoised using ANTs [72] then standardized using a linear scaling procedure based on values extracted from two anatomical masks located at the eyes and temporal muscles [75]. Myelination images were then obtained from the T1w/T2w ratio, and the average myelination intensity was calculated in each region by excluding outlier voxels (> 3SD).

Cortical surface reconstruction was performed using a FreeSurfer-based pipeline to generate white-matter and pial surfaces. Cortical regions were defined according to the CHARM atlas [74]. For each retained cortical ROI, cortical gray-matter volume, gray-matter thickness, and gray-matter surface area were extracted. Subcortical regions were parcellated using the SARM atlas [76]. Atlas labels were transformed from template space to individual subject space using the inverse registration transformation, and regional volumes were calculated based on voxel counts.

***Preprocessing rs-fMRI Data****.* Rs-fMRI data were preprocessed using AFNI [70]. EPI susceptibility distortion was corrected using reverse phase-encoding images. For each animal, the anatomical image was aligned to the EPI images to minimize interpolation-related changes to the EPI signals and to support subsequent anatomical registration. Slice-timing correction was then applied, followed by motion correction of the EPI volumes using 12 motion parameters. Co-registration to NMT space was achieved through the individual anatomical scans. Large transient signals were attenuated using AFNI 3dDespike. To improve the signal-to-noise ratio, EPI volumes were spatially smoothed with a 2-mm full-width at half-maximum (FWHM) Gaussian kernel. Voxel-wise signal intensities were scaled to yield a mean time-series value of 100 for each voxel. To reduce confounding effects, linear trends, head-motion parameters, and the average time series within the white matter (WM) and CSF masks were regressed out. WM and ventricular CSF nuisance masks were defined in the NMT template space and applied the same masks to both groups. Given the pronounced ventricular enlargement and anatomical distortion observed in the *ANK2* KO monkeys, we used anatomically constrained masks to minimize potential tissue misclassification during nuisance-signal extraction. Specifically, the WM mask was restricted to anatomically preserved frontoparietal white matter, corresponding to the cortical territories retained for rs-fMRI analysis, whereas the CSF mask was confined to ventricular CSF compartments. After resampling to each animal, all masks were visually inspected to confirm correct tissue localization, ensuring that the WM mask remained within white matter and that the CSF mask remained within ventricular CSF compartments. Mean WM and CSF signals were then extracted from these masks and included as nuisance regressors in the rs-fMRI preprocessing pipeline. No temporal bandpass filter was applied to the BOLD signals because neural information may be present outside the conventional 0.01–0.1 Hz frequency range, including higher-frequency components. Each time series was divided into five segments of 100 time points, and subsequent analyses were conducted separately for each segment.

***Functional connectivity (FC).*** FC was calculated as Pearson correlation coefficients between the mean time series of two brain nodes from 90 regions, resulting in a 90 × 90 matrix, which was then standardized as Z -scores using Fisher’s Z -transformation. The 90 regions (Table S3) were grouped into lobes (frontal, parietal) and subcortical nuclei for each hemisphere, creating a 6 × 6 connectivity matrix by averaging connectivity values within and between lobes, similar to our previous study [67]. These matrices were further categorized as left intra-hemispheric, right intra-hemispheric, and inter-hemispheric connections. Regional FC was indicated by FC density (FCD), which was calculated as the average of positive connections to the remaining 89 areas, with negative connections set to zero [77].

***Graph theory.*** The graph theory analysis, specifically whole-brain and nodal network analyses, were conducted using the GRETNA toolbox [78]. Whole-brain network was demonstrated by the small-world coefficient (σ), which reflected both global integration and local specialization. Nodal network metrics included the nodal clustering coefficient (NCp), an indicator of functional segregation, and nodal degree centrality (Dc), which quantifies the number of connections a node has and thus its importance within the network [79]. A range of sparsity thresholds from 0.20 to 0.50 (in 0.05 increments) was applied to ensure accurate estimation. Whole-brain and nodal metrics were computed at each threshold, and the area under the curve (AUC) was calculated for each metric [80].

***Brain–behavior correlation analysis.*** To explore the behavioral relevance of regional neuroimaging abnormalities, three representative behavioral outcomes were selected for correlation testing: working memory accuracy at B = 2 s in the spatial delayed response task, perseverative errors in the reversal learning task, and the cobra-minus-neutral retrieval latency difference in the emotional responsiveness test. Neuroimaging variables were restricted to measures showing significant group differences after FDR correction. Partial Spearman correlations were performed in animals with available paired behavioral and neuroimaging data, controlling for both species and genotype, with genotype included to reduce the potential influence of the overall KO-WT group difference on the observed associations.

**Physiological, hematological and lipidomics measurements**

Body weight, body length, head circumference, upper-limb length, and lower-limb length were recorded for each monkey at six time-points (2.3, 2.6, 2.8, 3.6, 4.4 and 7 years old), as described in our previous study [67]. Rectal temperature and heart rate were recorded simultaneously. Cardiac morphology and functions were determined by M-mode echocardiography using a veterinary ultrasound color doppler system (Mylab ClassC VET, Esaote, Italy) (Table S7). All measurements were conducted under light anesthesia maintained with ketamine hydrochloride (5 mg/kg, i.m., provided by Jiangsu Zhongmu Beikang Pharmaceutical Co. Ltd., China).

The fasting blood was collected from the monkey’s femoral vein for assessing hematological and ELISA assays. Briefly, serum was extracted from coagulants tube after centrifuged at 1489 g at 4 ℃ for 15 min and then was used for measuring blood biochemical parameters via integrated chemistry system (Dimension EXL 200, Siemens, Germany). We combined data for both monkey species together for hematological analysis, considering the physiological similarity between rhesus and cynomolgus monkeys. We expanded the sample size of WT group by 78 age-matched monkeys (58 rhesus and 20 cynomolgus monkeys) from our breeding colonies for blood biochemistry test (BCT) at the same measurement.

The serum cortisol and testosterone levels were measured using the QuicKey Pro macaque Cortisol ELISA Kit (Elabscience Biotechnology, E-OSEL-MK0002) and the monkey testosterone (T) ELISA kit (Shanghai Zhenke Biotechnolog, product No. ZK-12626), respectively, according to the manufacturers’ protocols. Each sample was measured in triplicate repetitions. EDTA-K2-anticoagulated whole blood samples were collected for measurement of complete blood count (CBC) at three developmental time points (age of 35, 47 and 53 months) (Table S7) via an automatic animal blood cell analyzer (BC2800-vet, Shenzhen Mindray Bio-Medical Electronics Co., Ltd., China).

We also performed targeted lipidomics analysis for cerebrospinal fluid (CSF) and serum samples from WT and KO monkeys. We pooled rhesus and cynomolgus monkeys into KO (*n* = 4) and age-matched WT animals (*n* = 17, including 11 rhesus and 6 cynomolgus monkeys) to maximize the sample size of *ANK2* KO genotype and WT controls to reduce individual differences. Serum samples were collected as described above and CSF samples were collected from the interspace between the last two lumbar with a sterilized 22-gauge puncture needle in monkeys under anesthesia with ketamine (10 mg/kg, i.m.). The CSF sample was centrifuged at 4 ℃, 3000 rpm for 15 min to separate supernatant, which was then immediately stored at −80 °C until analysis. All the lipids were extracted using the MTBE method, and lipid analyses were based on LC/MS, following the procedure described in our previous study [35]. Briefly, the analyses were performed using a UHPLC system (LC-30AD, Shimadzu) coupled with QTRAP MS (6500+, Sciex) at Shanghai Applied Protein Technology Co., Ltd. The analytes were separated using a HILIC column (Phenomenex, Luna NH2, 2.0 mm × 100 mm, 3 µm) and a C18 column (Phenomenex, Kinetex C18, 2.1 × 100 mm, 2.6 μm). The measured metabolites were quantified using Multi Quant or Analyst software.

**Peripheral blood sample collection, RNA extraction, and RNA sequencing**

For each monkey, 1 mL of venous blood was collected into EDTA-coated collection tubes. Blood samples were immediately transferred to an RNase-free laboratory and centrifuged at 3000 rpm for 10 min at room temperature to separate plasma. The remaining blood samples were incubated with red blood cell lysis buffer (Tiangen, China, RT122-01) at room temperature for 5 min to remove erythrocytes, followed by centrifugation at 4000 rpm at 4 ^o^C for 10 min. Leucocyte pellets were subjected to total RNA extraction by using the RNAeasy kit (TianGen, co.td.) according to the manufacturer’s instructions. The integrity and concentration were tested with NanoDrop biophotometer (Thermo Fisher Scientific).

For each monkey, 1 μg of RNA was used for RNA-sequencing. Sequencing libraries were generated using the NEBNextUltraTM RNA Library Prep kit for Illumina (NEB, USA) following the manufacturer’s recommendation and index codes were added to attribute sequence to each sample. The processed final library was sequenced on an Illumina Hiseq 4000 platform, and 150 bp paired-ends reads were generated. Raw reads were trimmed to remove sequencing adapters and low-quality reads using Trimmomatic 0.39 [81] The clean reads were then aligned to the corresponding reference genome (cynomolgus monkey: Macaca_fascicularis_5.0; rhesus monkeys: Mmul_8.0.1) using HISAT2 [82]. FeatureCounts was used to generate counts for known genes [83] and the differential expression analyses were performed using DESeq2 with gender as a covariate [84]. Given the genetic differences between the two macaque species and inter-individual variability, we employed a relatively lenient threshold for defining differentially expressed genes (DEGs), with genes showing |log2FoldChange| > 1 and an uncorrected *p* value < 0.05 considered as DEGs. Orthologous genes among cynomolgus monkey, rhesus monkey, and human were extracted from the Ensembl database (http://www.ensembl.org). Gene Ontology enrichment analysis of target gene sets was analyzed by DAVID 6.8 (https://david.ncifcrf.gov) [85]. Enriched terms with an FDR < 0.05 were considered statistically significant.

**Statistical analysis**

Data were expressed as mean ± standard error of the mean (S.E.M) and analyzed using the GraphPad Prism v10.50 (GraphPad Software, La Jolla, CA, USA) and Matlab. For all results, *p* values were uncorrected unless explicitly indicated as corrected *p* values in the results. After verifying data normality with the Shapiro-Wilk test, appropriate statistical methods were selected. A one-sample *t*-test was used in the SDR task to assess whether the mean B value in WT monkeys differed from the hypothetical value of *B* = 0 s, for all KO monkeys were *B* = 0s. The repeated-measures data including the five delay lengths in SDR task, two stages in VD/RL task and three phases in SIT were analyzed using a linear mixed-effects model (LMM), which included genotype and delay, stage or phase as fixed effects. Nonparametric Wilcoxon matched-pairs signed rank test was used for concerning the mean latency, ADI and trials before/after rubber cobra within group, as well as nonparametric Friedman test was used for concerning the latency across five sessions to compare the session effect between group in ER test. For the remaining behavioral and physiological data, normally distributed datasets were analyzed using Student’s *t* tests, whereas non-normally distributed data were analyzed with the nonparametric Mann-Whitney *U* test, due to non-repeated measurements. Specifically, multiple *t*-tests with the FDR correction were used for the 3D kinematic parameters. Statistical significance was set at FDR-corrected *p* < 0.05 for 3D kinematic parameters or uncorrected *p* <0.05 for other behavioral and physiological data.

For the analysis of MRI and fMRI data, we used a unified modeling framework across all brain regions, rather than selecting region-specific model families based on post hoc normality or variance tests. Specifically, generalized linear mixed models (GLMMs) with a Gaussian distribution and identity link function were used for the primary analyses, given that the dependent variables were continuous. Structural MRI and myelination differences were assessed using GLMMs at regional levels, with Hemisphere and Species included as random effects. For the global rs-fMRI metric, GLMMs were performed with Segment and Species as random effects. For lobe- and region-level rs-fMRI metrics (e.g., FC and graph theory metrics), GLMMs included Segment, Hemisphere, and Species as random effects. To improve transparency, we added a statistical summary table (Table S6) for the corrected and uncorrected *p* < 0.05 findings reported in the main and supplementary results.

The relevant distributional assumptions primarily concern the model residuals rather than the raw regional values. Shapiro-Wilk test was conducted assess normality, and Brown-Forsythe tests were conducted to evaluate homogeneity of variances.

Most findings showed clear evidence of residual normality. For findings whose residuals did not pass the Shapiro-Wilk test, we further inspected residual Q-Q plots, which did not indicate severe departures from the Gaussian mixed-model assumption. Only a small number of FDR-corrected findings showed evidence of variance heterogeneity. We performed additional subject-level Welch-based sensitivity analyses for these findings to validate the results. Specifically, repeated measurements within each animal were first averaged to obtain one subject-level value for each relevant region or metric, and Welch tests were then used to evaluate whether the corresponding effects remained robust under unequal-variance assumptions. The direction of the effects remained unchanged, and all tested effects remained significant or approximately significantly in the Welch-based sensitivity analyses, indicating that these findings were robust to unequal-variance assumptions. These results were listed in Table S6.

The FDR correction was applied within each analysis family across the applicable tested regions. Cortical-only MRI measures, such as cortical thickness and surface area, were corrected across the retained cortical regions of interest (ROIs, *n* = 39), while measures applicable to both cortical and subcortical regions were corrected across all retained ROIs (*n* = 45). Lobe-pair analyses were corrected across the tested lobe pairs (*n* = 6). Given the relatively small sample size, both corrected and uncorrected significant results were reported to provide a comprehensive view.

For the lipidomic analysis, we firstly constructed an orthogonal partial least squares-discriminate analysis (OPLS-DA) model and observed a limited reliability of this multivariate statistical model, validating by extremely low R2X, R2Y and Q2 in the 7-fold cross-validation and permutation tests. Therefore, we screened for significantly altered metabolites through univariate analyses. Significantly different lipid specie between the two groups were identified using the Mann–Whitney U test combined with fold change criteria, owing to the small sample size and large proportion of non-normal distributions. A metabolite was considered significantly different if it met the threshold of uncorrected *p* < 0.05 and had a fold change > 1.5 or < 2/3, on account for inter-individual variability. The Mann–Whitney U test was also applied to assess differences in chain length, lipid saturation, as well as comparisons at the category and class levels. Uncorrected *p* < 0.05 was also considered as significantly different.

**Fig. S1. Study design and timeline for longitudinal measurements.**

**Fig. S2. A profile of behaviors showing no significant difference between the giant *ANK2* knockout (KO) and wild-type (WT) monkeys.**

(A) The total score, sensory score and motor score in neurological functions. (B) Average vector magnitude count (VMC) at daytime determined from Actigraph. The sleep pattern was assessed by total sleep time and sleep efficiency. (C) Duration of exploring behaviors. (D) Mean retrieval duration, losing frequency and proportions of precisely retrieval in the vertical slot task. (E) The mean food-retrieval latency of the trial before or after the rubber snake emerged in the fear-induced emotional response test. (F-G) Representative footages showing the solitary behavior of rhesus KO monkey (T111) and WT monkey (WT) in a group-rearing condition (F), and a male KO rhesus monkey (T105) was reared alone in a single colony unit (G). (H) Passive grooming time, passive social interaction time and duration of social withdrawal during the group-reared condition. Student’s *t* test or Mann-Whitney U test for (A-D, H); Wilcoxon matched-pairs signed rank test for (E). In the violin plot in (A) (related to Fig. 1), the center line represents the median, the box indicates the interquartile range (IQR), and the whiskers extend to 1.5 times the IQR to illustrate the full data distribution. Other data were presented as mean ± S.E.M. KO group, *n* = 4 (including 2 rhesus and 2 cynomolgus monkeys); WT group, *n* = 10-11 (4-5 rhesus monkeys and 6 cynomolgus monkeys; one rhesus WT monkey died from intestinal obstruction at age 7).

**Fig. S3. Comparisons of cognitive performance between cynomolgus (Cyn) and rhesus (Rhe) monkeys.**

(A) Percentage of correct choice at *B*=2s in SDR task. (B) Number of corrections across five delays. (C-D) Number of trials to criteria, square of errors to criteria and error rate in VD stage (C) and RL stage (D) during the VD/RL task. (E) Perseverative errors after reversal during reversal learning task. (F) Manual training trials to reach criterion in operant learning task. Filled black circles represent KO monkeys, whereas open circles represent WT individuals. Data were presented as mean ± S.E.M. Mann-Whitney U test for (A) and (F); Student’s *t* test for (E); Linear mixed-effects models for (B-D).

**Fig S4. MRI/fMRI cortical and subcortical analysis mask.**

Representative examples of cortical and subcortical masks used for MRI/fMRI analysis, overlaid on the T1-weighted images (monkey T87) showing the most pronounced ventricular abnormality. The masks illustrate the cortical and subcortical regions included in the quantitative analysis. Colors on the atlas denote distinct brain regions.

**Fig. S5. Changes in gray matter volume and surface area in the giant *ANK2* knockout (KO) monkeys.**

(A) Gray matter volume of regions with differences between KO and WT monkeys. (B) Visualization of the regions in (A) on a monkey brain template. (C) Gray matter surface area of regions with differences between KO and WT monkeys. (D) Visualization of the regions in (C) on a monkey template. Regions surviving FDR correction and regions showing group differences at the uncorrected threshold are displayed. * *p* < 0.05 **, *p* < 0.01 ***, *p* < 0.001, KO versus WT; generalized linear mixed models corrected by the false discovery rate (FDR) method. Red asterisks denoted false discovery rate (FDR)-corrected *p* values; black asterisks denoted uncorrected *p* values. Data were presented as mean ± S.E.M. This figure is related to Fig. 4. KO group, *n* = 4 (including 2 rhesus and 2 cynomolgus monkeys); WT group, *n* = 11 (including 5 rhesus and 6 cynomolgus monkeys). The brain regions were defined in Table S3.

**Fig. S6.** **Changes in degree centrality (Dc) and nodal clustering coefficient (NCp) in the giant *ANK2* knockout (KO) monkeys.**

(A) Cortical regions with differences in Dc between KO and WT monkeys. (B) Subcortical regions with differences in Dc. (C) Visualization of the regions in (A) on a monkey brain template. (D) Cortical regions with differences in NCp. (E) Enhanced NCp of Hy from subcortical structure. (F) Visualization of the regions in (D) on a monkey brain template. Regions that survived FDR correction are marked with red asterisks (FDR-corrected *p* values), whereas regions showing group differences only at the uncorrected threshold are marked by black asterisks (uncorrected *p* values). * *p* < 0.05 **, *p* < 0.01 ***, *p* < 0.001, KO versus WT; generalized linear mixed models corrected by the false discovery rate (FDR) method. Data were presented as mean ± S.E.M. This figure is related to Fig. 4. KO group, *n* = 4 (including 2 rhesus and 2 cynomolgus monkeys); WT group, *n* = 11 (including 5 rhesus and 6 cynomolgus monkeys). The brain regions were defined in Table S3.

**Fig. S7. Associations between regional neuroimaging measures and working memory performance.**

(A-B) Partial Spearman rank correlations of working memory accuracy at *B* = 2 s with cortical thickness in area 24a/b prime (A) and putamen degree centrality (Dc) (B), controlling for species and genotype. Each point represents an individual monkey with available paired behavioral and neuroimaging data (*n* = 13). MF: *Macaca fascicularis*; MM: *Macaca mulatta;* KO, knockout; WT, wild-type.

**Fig. S8.** **No significant differences in developmental trajectory between the giant *ANK2* knockout (KO) and wild-type (WT) monkeys**.

(A-E) Change of body weight (A), head circumference (B), body length (C), upper-limb length (D), and lower-limb length (E) across age in the KO and WT rhesus monkeys. (F-G) Heart rate (F) and (G) rectal temperatures in the KO and WT rhesus monkeys. (H-L) Change of body weight (H), head circumference (I), body length (J), upper-limb length (K), and lower-limb length (L) across age in the KO and WT cynomolgus monkeys. (M-N) Heart rate (M) and rectal temperature (N) in the KO and WT cynomolgus monkeys. Data were presented as mean ± S.E.M. Each data point denotes a single measure for an individual, total six measures. (A-G): KO rhesus monkey (KOR) group, n=2; WT rhesus monkey (WTR) group, n=5; (H-N): KO cynomolgus monkey (KOC) group, *n* = 2; WT cynomolgus monkey (WTC) group, *n* = 6. Statistical analyses were performed by Student’s *t* test or Mann-Whitney U test.

**Fig. S9. Comparison of lipidomic species in cerebrospinal fluid (CSF) from the giant *ANK2* knockout (KO) and wild-type (WT) monkeys.**

(A) The total lipid class and lipid species determined by LC-MC from CSF (left panel) and serum (right panel). The number on the top of each bar refers to the total lipid species in the corresponding class. (B) Violin plot showing no significant differences in the concentrations of total lipids and each lipid category between the KO and WT monkeys. (C) Bar plot showing total unsaturated TG (up panel) and unsaturated TG to saturated TG ratio (down panel) between KO and WT monkeys. (D) Indicative features of membrane fluidity levels [38] for each individual. The plot showed six features (from left to right): saturated GP to total GP ratio, saturated PC to total PC ratio, saturated PE family to total PE family ratio, concentrations of total unsaturated GP, PC and PE family. The PE family includes PE, PE-O and PE-P. The former three features were negatively correlated with fluidity, while the later three were positively correlated with fluidity. No differences were found in each feature between the two groups, indicative of unchanged membrane fluidity in KO monkeys. **, *p* < 0.01; KO versus WT; Mann-Whitney U test. Data in (B) were presented as the median and interquartile range (IQR), while data in other figure sections were presented as mean ± S.E.M. This figure is related to Fig. 5. KO group, *n* = 4 (including 2 rhesus and 2 cynomolgus monkeys); WT group, *n* = 17 (including 11 rhesus monkeys and 6 cynomolgus monkeys).

**Fig. S10**. **Expression (A) and differential expression (B) of genes in peripheral blood between cynomolgus monkeys and rhesus monkeys.** Mean gene expression (log2 normalized) and differential expression (represented by log2FoldChange) of 9182 human orthologous genes in two monkey species were used for analyses. Correlation coefficients and *p* values were calculated using Pearson’s correlation.

**Table S1 Characteristics and comparison of 46 kinematics in rhesus monkeys (*Macaca mulatta*) between the giant *ANK2* knockout (KO) and wild-type (WT)**

| **Parameter** | **WT (*n* = 5)** | **KO (*n* = 2)** | ***t*** | **Corrected *p* value** |
| --- | --- | --- | --- | --- |
| **Movement speed** **(mm/s)** | |  |  |  |
| Nose | 301.02 ± 63.63 | 756.64 ± 30.57 | 4.353 | 0.005 ** |
| Left eye | 280.96 ± 63.28 | 756.04 ± 34.67 | 4.544 | 0.005 ** |
| Right eye | 278.73 ± 63.74 | 741.6 ± 30.15 | 4.417 | 0.005 ** |
| Head | 263.81 ± 62.33 | 717.13 ± 38.69 | 4.378 | 0.005 ** |
| Neck | 237.6 ± 56.34 | 635.52 ± 20.91 | 4.320 | 0.005 ** |
| Left shoulder | 260.48 ± 50.99 | 572.41 ± 21.72 | 3.730 | 0.010 ** |
| Right shoulder | 261.23 ± 51.92 | 591.16 ± 17.62 | 3.892 | 0.009 ** |
| Left elbow | 340.82 ± 45.82 | 586.41 ± 22.44 | 3.257 | 0.014 * |
| Right elbow | 338.8 ± 45.73 | 571.18 ± 20.39 | 3.096 | 0.016 * |
| Left wrist | 461.34 ± 55.8 | 769.39 ± 54.01 | 3.213 | 0.014 * |
| Right wrist | 456.39 ± 50.97 | 726.56 ± 24.51 | 3.222 | 0.014 * |
| Left limb | 234.61 ± 30.86 | 395.71 ± 35.48 | 2.971 | 0.017 * |
| Right limb | 240.97 ± 31.99 | 400.35 ± 55.47 | 2.598 | 0.027 * |
| Left knee | 260.78 ± 30.97 | 384.84 ± 18.89 | 2.413 | 0.037 * |
| Right knee | 269.05 ± 32.45 | 379.59 ± 23.51 | 2.033 | 0.068 |
| Left ankle | 268.13 ± 36.66 | 401.49 ± 14.09 | 2.223 | 0.049 * |
| Right ankle | 273.5 ± 31.88 | 426.27 ± 31.95 | 2.777 | 0.021 * |
| Back | 222.53 ± 32.87 | 381.95 ± 17.84 | 2.936 | 0.017 * |
| Tail root | 232.05 ± 30.57 | 424.8 ± 73.64 | 2.922 | 0.017 * |
| Middle tail | 265.08 ± 31.83 | 576.6 ± 140.74 | 3.181 | 0.014 * |
| Tail tip | 368.24 ± 40.42 | 784.25 ± 207.83 | 2.986 | 0.017 * |
| **Movement intensity (AU)** | |  |  |  |
| Nose | 11.88 ± 2.59 | 31.08 ± 2.21 | 4.377 | 0.005 * |
| Left eye | 11.08 ± 2.58 | 30.84 ± 2.1 | 4.539 | 0.005 * |
| Right eye | 11 ± 2.59 | 30.4 ± 2.16 | 4.427 | 0.005 * |
| Head | 10.4 ± 2.54 | 29.15 ± 2.35 | 4.311 | 0.005 ** |
| Neck | 9.57 ± 2.29 | 25.62 ± 1.4 | 4.226 | 0.005 ** |
| Left shoulder | 10.21 ± 2.05 | 22.92 ± 1.37 | 3.714 | 0.010 ** |
| Right shoulder | 10.25 ± 2.1 | 23.47 ± 1.1 | 3.826 | 0.009 ** |
| Left elbow | 13.34 ± 1.77 | 23.83 ± 1.19 | 3.542 | 0.012 * |
| Right elbow | 13.25 ± 1.78 | 23 ± 1.07 | 3.304 | 0.014 * |
| Left wrist | 19.24 ± 2.28 | 31.87 ± 2.1 | 3.244 | 0.014 * |
| Right wrist | 19.08 ± 2.03 | 30.27 ± 1.44 | 3.294 | 0.014 * |
| Left limb | 9.34 ± 1.15 | 15.59 ± 1.57 | 3.004 | 0.017 * |
| Right limb | 9.63 ± 1.2 | 15.81 ± 2.17 | 2.663 | 0.026 * |
| Left knee | 9.92 ± 1.08 | 14.97 ± 0.76 | 2.777 | 0.021 * |
| Right knee | 10.27 ± 1.13 | 14.68 ± 0.84 | 2.329 | 0.042 * |
| Left ankle | 10.38 ± 1.38 | 15.97 ± 0.42 | 2.488 | 0.033 * |
| Right ankle | 10.56 ± 1.2 | 16.67 ± 1.07 | 2.982 | 0.017 * |
| Back | 8.78 ± 1.17 | 14.51 ± 0.76 | 2.933 | 0.017 * |
| Tail root | 9.37 ± 1.11 | 17.66 ± 3.19 | 3.180 | 0.014 * |
| Middle tail | 10.58 ± 1.14 | 24.19 ± 6.16 | 3.327 | 0.014 * |
| Tail tip | 14.54 ± 1.47 | 33.29 ± 9.13 | 3.178 | 0.014 * |
| **Distance per frame (mm)** | 5.6 ± 1.05 | 11.43 ± 0.44 | 3.387 | 0.014 * |
| **Body length (mm)** | 421.39 ± 13.04 | 443.72 ± 21.33 | 0.907 | 0.391 |
| **Body height (mm)** | 198.28 ± 12.8 | 255.76 ± 12.59 | 2.609 | 0.027 * |
| **Body angle (**^o^ **)** | 90.34 ± 0.05 | 90.36 ± 0.05 | 0.248 | 0.808 |

Note: Data were presented as mean ± S.E. M. KO, giant *ANK2* knockout rhesus monkey; WT, wild-type rhesus monkey; AU, arbitrary units. *, corrected *p* < 0.05, **, corrected *p* < 0.01; multiple *t* tests with false discovery rate (FDR) correction.

**Table S2 Description of** **the kinematic parameters and locomotive behaviors in 3D-motion analysis**

| **Assessed parameters** | **Variables** | **Variable types** | **Definitions** |
| --- | --- | --- | --- |
| **Kinematic parameters** | Movement speed | Velocity | The planar (x and y-plane) movement velocity per frame for 21 body key points of the monkey. |
|  | Movement intensity | Velocity | The 3D movement velocity of 21 body key points, calculated as the 3D displacement per frame. |
|  | Movement distance | Distance | The cumulative sum of planar (x and y-plane) displacement per frame. |
|  | Body length | Length | The linear distance between nose and tail root, calculated as the Euclidean distance between the point of nose and root tail. |
|  | Body height | Height | The average elevation of the monkey, computed by subtracting the mean z-coordinate of two fore wrists and two hind ankles from the z-coordinate of the back. |
|  | Body angle | Angle | The angular configuration formed by neck, back, and tail root, derived from the angle between these three points. |
| **Apparent displacement (AD) behaviors** | Climb | Proportion of duration | Monkey ascends or moves horizontally with discernible velocity |
|  | Fall | Proportion of duration | Monkey moves downward from the elevated position with discernible velocity |
|  | Turn left | Proportion of duration | Monkey executes a leftward rotation in the lower cage region with rapid movement |
|  | Turn right | Proportion of duration | Monkey executes rightward rotation in the lower cage region with rapid movement |
|  | Walk | Proportion of duration | Monkey ambulates linearly in the lower cage region with rapid movement |
|  | Jump | Proportion of duration | Monkey propels upward with significant height change and instantaneous high velocity |
| **Non-apparent displacement (NAD) behaviors** | Crouch | Proportion of duration | Monkey maintains a stationary curled posture in the lower cage region |
|  | Stand up | Proportion of duration | Monkey elevates its body with at least one hind limb contacting the ground at low velocity |
|  | Sit | Proportion of duration | Monkey maintains a flexed hindlimb posture in the lower cage region at low velocity |
|  | Look left | Proportion of duration | Monkey orients head leftward (>25° deviation) in the lower cage region at low velocity |
|  | Look right | Proportion of duration | Monkey orients head rightward (>25° deviation) in the lower cage region at low velocity |
|  | Head rise | Proportion of duration | Monkey elevates its head in the lower cage region at low velocity |
|  | Head lower | Proportion of duration | Monkey depresses its head in the lower cage region at low velocity |
|  | Hang | Proportion of duration | Monkey clings to cage walls or ceiling in an elevated position with minimal movement velocity |

**Table S3 The abbreviations and region names of brain areas for neuroimaging analyses**

| **Abbreviation** | **Region name** | **Abbreviation** | **Region name** |
| --- | --- | --- | --- |
| Acb | accumbens | area 7op | parietal operculum |
| AIP | anterior intraparietal area | area 8A | periarcuate area 8A (Frontal Eye Fields) |
| Amy | amygdala | area 8B | area 8B |
| area 10 | frontal pole | area 9 | area 9 |
| area 11 | area 11 | areas 1-2 | areas 1-2 |
| area 12m/o | medial and orbital area 12 | G | gustatory cortex |
| area 12r/l | rostral and lateral area 12 | Hy | hypothalamus |
| area 13 | area 13 | Iam/Iapm | medial agranular insular region |
| area 14 | gyrus rectus | lat Ia | lateral agranular insular region |
| area 23 | area 23 | LIP | lateral intraparietal area |
| area 24a/b | areas 24a and 24b | LVPal | lateral and ventral pallium |
| area 24a/b prime | areas 24a' and 24b' | M1 | primary motor cortex |
| area 24c | area 24c | OLF | primary olfactory cortex |
| area 24c prime | area 24c' | Pd | pallidum |
| area 25 | subgenual cortex | PEa | area PEa |
| area 31 | area 31 | PMd | dorsal premotor cortex |
| area 32 | area 32 | PMv | ventral premotor cortex |
| area 3a/b | areas 3a and 3b | PrCO | precentral operular area |
| area 44 | area 44 | preSMA | presupplementary motor area |
| area 45 | area 45 | Pu | putamen |
| area 46d | dorsal area 46 | SII | secondary somatosensory cortex |
| area 46v/f | ventral and fundus portion of area 46 | SMA | supplementary motor area |
| area 5d | dorsal area 5 |  |  |

**Table S4 Comparison of the structural and functional MRI parameters in cortical regions between the giant *ANK2* knockout (KO) and wild-type (WT) monkeys**

| **Parameter** | **Region** | **WT (*n* = 11)** | **KO (*n* = 4)** | ***p* value** | **Corrected *p* value** |
| --- | --- | --- | --- | --- | --- |
| **Myelination** | area 32 | 0.104 ± 0.002 | 0.103 ± 0.008 | 0.775 | 0.843 |
|  | area 25 | 0.106 ± 0.002 | 0.111 ± 0.006 | 0.215 | 0.448 |
|  | area 24a/b | 0.104 ± 0.002 | 0.107 ± 0.010 | 0.484 | 0.751 |
|  | area 24c | 0.104 ± 0.002 | 0.104 ± 0.010 | 0.963 | 0.985 |
|  | area 24a/b prime | 0.123 ± 0.002 | 0.130 ± 0.009 | 0.241 | 0.472 |
|  | area 24c prime | 0.126 ± 0.002 | 0.129 ± 0.009 | 0.757 | 0.843 |
|  | area 10 | 0.107 ± 0.002 | 0.109 ± 0.007 | 0.527 | 0.774 |
|  | area 11 | 0.112 ± 0.002 | 0.119 ± 0.007 | 0.136 | 0.418 |
|  | area 13 | 0.117 ± 0.002 | 0.123 ± 0.006 | 0.208 | 0.448 |
|  | area 12m/o | 0.117 ± 0.002 | 0.125 ± 0.006 | 0.150 | 0.418 |
|  | Iam/Iapm | 0.111 ± 0.003 | 0.123 ± 0.004 | **0.040*** | 0.301 |
|  | lat Ia | 0.108 ± 0.002 | 0.114 ± 0.006 | 0.186 | 0.440 |
|  | OLF | 0.116 ± 0.002 | 0.119 ± 0.006 | 0.550 | 0.774 |
|  | G | 0.126 ± 0.002 | 0.124 ± 0.007 | 0.658 | 0.834 |
|  | PrCO | 0.113 ± 0.002 | 0.115 ± 0.007 | 0.699 | 0.834 |
|  | area 8A | 0.141 ± 0.003 | 0.148 ± 0.010 | 0.365 | 0.619 |
|  | area 8B | 0.127 ± 0.002 | 0.131 ± 0.010 | 0.574 | 0.782 |
|  | area 9 | 0.109 ± 0.002 | 0.114 ± 0.011 | 0.407 | 0.654 |
|  | area 46d | 0.119 ± 0.002 | 0.124 ± 0.009 | 0.313 | 0.563 |
|  | area 46v/f | 0.119 ± 0.002 | 0.122 ± 0.010 | 0.547 | 0.774 |
|  | area 12r/l | 0.117 ± 0.002 | 0.123 ± 0.008 | 0.267 | 0.501 |
|  | area 45 | 0.136 ± 0.002 | 0.139 ± 0.009 | 0.616 | 0.815 |
|  | area 44 | 0.127 ± 0.002 | 0.126 ± 0.009 | 0.915 | 0.957 |
|  | M1 | 0.169 ± 0.003 | 0.184 ± 0.008 | **0.036*** | 0.301 |
|  | PMd | 0.150 ± 0.003 | 0.162 ± 0.009 | 0.098 | 0.418 |
|  | PMv | 0.139 ± 0.002 | 0.141 ± 0.009 | 0.704 | 0.834 |
|  | preSMA | 0.116 ± 0.002 | 0.118 ± 0.011 | 0.724 | 0.836 |
|  | SMA | 0.134 ± 0.002 | 0.143 ± 0.009 | 0.166 | 0.418 |
|  | area 3a/b | 0.163 ± 0.003 | 0.173 ± 0.008 | 0.167 | 0.418 |
|  | areas 1-2 | 0.155 ± 0.003 | 0.164 ± 0.009 | 0.219 | 0.448 |
|  | SII | 0.136 ± 0.003 | 0.138 ± 0.008 | 0.787 | 0.843 |
|  | area 5d | 0.141 ± 0.003 | 0.151 ± 0.008 | 0.161 | 0.418 |
|  | PEa | 0.151 ± 0.003 | 0.163 ± 0.008 | 0.114 | 0.418 |
|  | AIP | 0.150 ± 0.003 | 0.161 ± 0.009 | 0.162 | 0.418 |
|  | LIP | 0.145 ± 0.003 | 0.156 ± 0.007 | 0.119 | 0.418 |
|  | area 7op | 0.149 ± 0.004 | 0.152 ± 0.008 | 0.667 | 0.834 |
|  | area 31 | 0.132 ± 0.003 | 0.144 ± 0.007 | 0.057 | 0.365 |
|  | area 23 | 0.129 ± 0.003 | 0.134 ± 0.007 | 0.371 | 0.619 |
| **Gray matter volume** | area 32 | 85.261 ± 5.466 | 73.469 ± 8.352 | **0.006**** | 0.090 |
|  | area 25 | 45.886 ± 2.888 | 42.094 ± 5.130 | 0.171 | 0.649 |
|  | area 24a/b | 96.653 ± 8.782 | 66.781 ± 11.665 | **0.001***** | **0.024*** |
|  | area 24c | 176.290 ± 10.511 | 182.922 ± 20.026 | 0.788 | 0.959 |
|  | area 24a/b prime | 93.455 ± 6.401 | 83.516 ± 11.609 | 0.099 | 0.555 |
|  | area 24c prime | 116.369 ± 6.005 | 120.063 ± 10.506 | 0.789 | 0.959 |
|  | area 10 | 187.903 ± 6.809 | 188.859 ± 27.410 | 0.924 | 1.000 |
|  | area 11 | 111.358 ± 7.548 | 107.188 ± 16.981 | 0.510 | 0.919 |
|  | area 13 | 367.256 ± 26.427 | 345.625 ± 40.894 | 0.232 | 0.653 |
|  | area 12m/o | 145.864 ± 9.449 | 135.234 ± 11.315 | 0.154 | 0.649 |
|  | Iam/Iapm | 24.080 ± 1.858 | 25.781 ± 3.229 | 0.625 | 0.959 |
|  | lat Ia | 154.858 ± 9.848 | 163.969 ± 21.750 | 0.645 | 0.959 |
|  | OLF | 49.693 ± 2.530 | 51.172 ± 5.086 | 0.848 | 0.978 |
|  | G | 44.369 ± 2.824 | 47.453 ± 5.473 | 0.510 | 0.919 |
|  | PrCO | 78.898 ± 4.542 | 74.219 ± 9.696 | 0.386 | 0.875 |
|  | area 8A | 76.830 ± 4.764 | 67.688 ± 11.659 | 0.061 | 0.459 |
|  | area 8B | 203.665 ± 9.499 | 211.016 ± 24.101 | 0.753 | 0.959 |
|  | area 9 | 240.983 ± 14.147 | 227.406 ± 24.689 | 0.208 | 0.649 |
|  | area 46d | 209.489 ± 11.645 | 205.391 ± 28.589 | 0.589 | 0.959 |
|  | area 46v/f | 268.483 ± 14.847 | 242.344 ± 25.202 | 0.076 | 0.489 |
|  | area 12r/l | 68.665 ± 7.496 | 59.953 ± 16.609 | 0.408 | 0.875 |
|  | area 45 | 144.261 ± 8.012 | 134.281 ± 13.101 | 0.179 | 0.649 |
|  | area 44 | 44.131 ± 2.237 | 43.609 ± 6.685 | 0.727 | 0.959 |
|  | M1 | 693.091 ± 35.158 | 665.438 ± 59.595 | 0.281 | 0.743 |
|  | PMd | 696.773 ± 40.408 | 766.594 ± 88.381 | 0.216 | 0.649 |
|  | PMv | 586.767 ± 27.591 | 595.156 ± 74.783 | 0.955 | 1.000 |
|  | preSMA | 77.114 ± 4.505 | 75.250 ± 9.696 | 0.480 | 0.919 |
|  | SMA | 154.989 ± 8.686 | 142.906 ± 15.368 | 0.997 | 1.000 |
|  | area 3a/b | 240.068 ± 12.301 | 240.375 ± 23.068 | 0.828 | 0.978 |
|  | areas 1-2 | 623.364 ± 30.926 | 601.781 ± 53.214 | 0.330 | 0.825 |
|  | SII | 264.710 ± 12.337 | 304.172 ± 42.663 | 0.113 | 0.563 |
|  | area 5d | 205.438 ± 10.945 | 196.906 ± 27.705 | 0.481 | 0.919 |
|  | PEa | 184.818 ± 8.676 | 172.938 ± 15.310 | 0.204 | 0.649 |
|  | AIP | 46.403 ± 2.268 | 45.906 ± 4.902 | 0.731 | 0.959 |
|  | LIP | 252.426 ± 12.701 | 228.719 ± 25.483 | 0.054 | 0.459 |
|  | area 7op | 170.403 ± 9.010 | 169.844 ± 18.770 | 0.689 | 0.959 |
|  | area 31 | 38.244 ± 3.260 | 41.656 ± 4.828 | 0.536 | 0.928 |
|  | area 23 | 293.006 ± 16.834 | 299.531 ± 28.876 | 0.972 | 1.000 |
| **Thickness** | area 32 | 2.012 ± 0.033 | 1.867 ± 0.073 | **0.019*** | 0.147 |
|  | area 25 | 1.864 ± 0.027 | 1.706 ± 0.046 | **0.003**** | **0.033*** |
|  | area 24a/b | 2.011 ± 0.053 | 1.521 ± 0.114 | **<0.001***** | **<0.001***** |
|  | area 24c | 1.982 ± 0.028 | 2.218 ± 0.064 | **0.001***** | **0.007**** |
|  | area 24a/b prime | 1.620 ± 0.041 | 1.362 ± 0.105 | **<0.001***** | **0.007**** |
|  | area 24c prime | 1.786 ± 0.019 | 1.760 ± 0.027 | 0.481 | 0.794 |
|  | area 10 | 1.652 ± 0.028 | 1.605 ± 0.090 | 0.314 | 0.757 |
|  | area 11 | 1.525 ± 0.039 | 1.553 ± 0.111 | 0.822 | 0.958 |
|  | area 13 | 1.946 ± 0.029 | 1.904 ± 0.071 | 0.408 | 0.757 |
|  | area 12m/o | 1.960 ± 0.049 | 1.975 ± 0.071 | 0.949 | 1.000 |
|  | Iam/Iapm | 1.669 ± 0.066 | 1.748 ± 0.091 | 0.536 | 0.809 |
|  | lat Ia | 2.124 ± 0.057 | 2.120 ± 0.095 | 0.816 | 0.958 |
|  | OLF | 1.854 ± 0.052 | 1.912 ± 0.090 | 0.589 | 0.851 |
|  | G | 1.666 ± 0.031 | 1.738 ± 0.042 | 0.217 | 0.705 |
|  | PrCO | 2.313 ± 0.050 | 2.353 ± 0.045 | 0.685 | 0.954 |
|  | area 8A | 1.900 ± 0.021 | 1.894 ± 0.050 | 0.836 | 0.958 |
|  | area 8B | 2.091 ± 0.023 | 2.128 ± 0.030 | 0.391 | 0.757 |
|  | area 9 | 2.132 ± 0.032 | 2.212 ± 0.040 | 0.180 | 0.666 |
|  | area 46d | 1.735 ± 0.021 | 1.781 ± 0.031 | 0.259 | 0.757 |
|  | area 46v/f | 1.542 ± 0.017 | 1.592 ± 0.041 | 0.188 | 0.666 |
|  | area 12r/l | 1.897 ± 0.034 | 1.783 ± 0.136 | 0.161 | 0.666 |
|  | area 45 | 1.958 ± 0.020 | 1.975 ± 0.044 | 0.725 | 0.958 |
|  | area 44 | 1.731 ± 0.022 | 1.784 ± 0.078 | 0.383 | 0.757 |
|  | M1 | 2.185 ± 0.021 | 2.099 ± 0.056 | 0.061 | 0.397 |
|  | PMd | 2.394 ± 0.021 | 2.410 ± 0.044 | 0.751 | 0.958 |
|  | PMv | 2.163 ± 0.020 | 2.193 ± 0.056 | 0.540 | 0.809 |
|  | preSMA | 2.204 ± 0.029 | 2.274 ± 0.073 | 0.285 | 0.757 |
|  | SMA | 2.251 ± 0.032 | 2.213 ± 0.092 | 0.489 | 0.794 |
|  | area 3a/b | 1.332 ± 0.018 | 1.332 ± 0.025 | 0.989 | 1.000 |
|  | areas 1-2 | 1.725 ± 0.020 | 1.694 ± 0.039 | 0.349 | 0.757 |
|  | SII | 1.861 ± 0.021 | 1.866 ± 0.059 | 0.968 | 1.000 |
|  | area 5d | 1.855 ± 0.030 | 1.859 ± 0.072 | 0.981 | 1.000 |
|  | PEa | 1.677 ± 0.027 | 1.633 ± 0.046 | 0.378 | 0.757 |
|  | AIP | 1.563 ± 0.022 | 1.491 ± 0.053 | 0.146 | 0.666 |
|  | LIP | 1.680 ± 0.021 | 1.649 ± 0.044 | 0.374 | 0.757 |
|  | area 7op | 1.649 ± 0.021 | 1.591 ± 0.032 | 0.075 | 0.419 |
|  | area 31 | 1.881 ± 0.046 | 1.910 ± 0.118 | 0.819 | 0.958 |
|  | area 23 | 1.648 ± 0.018 | 1.677 ± 0.046 | 0.488 | 0.794 |
| **Surface area** | area 32 | 42.625 ± 2.347 | 38.406 ± 3.139 | **0.027*** | 0.198 |
|  | area 25 | 27.341 ± 1.689 | 28.375 ± 3.298 | 0.825 | 0.919 |
|  | area 24a/b | 57.000 ± 3.997 | 48.063 ± 4.919 | **0.004**** | 0.100 |
|  | area 24c | 69.421 ± 4.587 | 64.906 ± 8.223 | 0.090 | 0.320 |
|  | area 24a/b prime | 68.330 ± 3.404 | 67.750 ± 6.039 | 0.674 | 0.874 |
|  | area 24c prime | 48.386 ± 2.552 | 55.625 ± 5.643 | **0.036*** | 0.200 |
|  | area 10 | 152.636 ± 3.731 | 150.906 ± 15.551 | 0.745 | 0.888 |
|  | area 11 | 82.932 ± 4.028 | 75.719 ± 7.259 | 0.068 | 0.296 |
|  | area 13 | 193.784 ± 12.057 | 186.656 ± 16.673 | 0.269 | 0.548 |
|  | area 12m/o | 88.784 ± 4.556 | 82.531 ± 6.249 | 0.197 | 0.521 |
|  | Iam/Iapm | 14.261 ± 0.681 | 15.313 ± 1.733 | 0.372 | 0.580 |
|  | lat Ia | 71.523 ± 3.044 | 76.969 ± 8.478 | 0.306 | 0.548 |
|  | OLF | 29.352 ± 1.024 | 29.219 ± 2.808 | 0.816 | 0.919 |
|  | G | 15.034 ± 0.968 | 15.469 ± 2.129 | 0.951 | 0.976 |
|  | PrCO | 43.205 ± 1.731 | 40.219 ± 4.901 | 0.266 | 0.548 |
|  | area 8A | 58.261 ± 3.394 | 49.469 ± 7.944 | **0.017*** | 0.169 |
|  | area 8B | 102.557 ± 4.457 | 102.688 ± 11.204 | 0.752 | 0.888 |
|  | area 9 | 144.080 ± 7.545 | 131.531 ± 12.492 | **0.031*** | 0.198 |
|  | area 46d | 151.705 ± 7.899 | 145.125 ± 18.889 | 0.301 | 0.548 |
|  | area 46v/f | 161.943 ± 7.906 | 141.781 ± 11.519 | **0.009**** | 0.120 |
|  | area 12r/l | 50.625 ± 4.593 | 43.969 ± 9.874 | 0.280 | 0.548 |
|  | area 45 | 88.546 ± 4.394 | 82.313 ± 6.299 | 0.152 | 0.455 |
|  | area 44 | 14.773 ± 0.689 | 14.188 ± 1.912 | 0.474 | 0.684 |
|  | M1 | 349.784 ± 14.900 | 346.188 ± 23.394 | 0.502 | 0.699 |
|  | PMd | 327.682 ± 17.010 | 357.438 ± 35.902 | 0.200 | 0.521 |
|  | PMv | 322.864 ± 12.532 | 320.281 ± 32.075 | 0.638 | 0.858 |
|  | preSMA | 44.534 ± 2.387 | 41.094 ± 5.136 | 0.101 | 0.327 |
|  | SMA | 88.534 ± 4.109 | 82.500 ± 7.000 | **0.005**** | 0.100 |
|  | area 3a/b | 173.830 ± 8.953 | 174.844 ± 14.853 | 0.872 | 0.919 |
|  | areas 1-2 | 451.386 ± 18.514 | 438.781 ± 29.643 | 0.336 | 0.570 |
|  | SII | 146.705 ± 4.872 | 164.094 ± 17.169 | 0.080 | 0.314 |
|  | area 5d | 146.261 ± 6.855 | 137.125 ± 15.347 | 0.247 | 0.548 |
|  | PEa | 120.364 ± 4.628 | 116.875 ± 6.549 | 0.395 | 0.592 |
|  | AIP | 21.580 ± 1.199 | 23.781 ± 2.293 | 0.309 | 0.548 |
|  | LIP | 176.830 ± 7.005 | 163.969 ± 14.237 | **0.045*** | 0.217 |
|  | area 7op | 98.375 ± 4.675 | 100.938 ± 9.354 | 0.869 | 0.919 |
|  | area 31 | 23.932 ± 1.951 | 27.844 ± 4.904 | 0.357 | 0.580 |
|  | area 23 | 168.761 ± 9.318 | 168.156 ± 14.484 | 0.695 | 0.874 |
| **Functional connectivity density (FCD)** | area 32 | 0.082 ± 0.003 | 0.071 ± 0.003 | 0.229 | 0.479 |
|  | area 25 | 0.064 ± 0.002 | 0.054 ± 0.003 | 0.068 | 0.305 |
|  | area 24a/b | 0.102 ± 0.004 | 0.072 ± 0.004 | **0.010**** | 0.088 |
|  | area 24c | 0.125 ± 0.005 | 0.104 ± 0.005 | 0.142 | 0.400 |
|  | area 24a/b prime | 0.108 ± 0.005 | 0.096 ± 0.005 | 0.366 | 0.514 |
|  | area 24c prime | 0.125 ± 0.005 | 0.129 ± 0.006 | 0.857 | 0.857 |
|  | area 10 | 0.100 ± 0.005 | 0.078 ± 0.005 | **0.046*** | 0.259 |
|  | area 14 | 0.075 ± 0.003 | 0.061 ± 0.003 | **0.036*** | 0.234 |
|  | area 11 | 0.090 ± 0.004 | 0.079 ± 0.005 | 0.259 | 0.485 |
|  | area 13 | 0.105 ± 0.004 | 0.084 ± 0.005 | 0.108 | 0.348 |
|  | area 12m/o | 0.093 ± 0.005 | 0.079 ± 0.004 | 0.245 | 0.479 |
|  | Iam/Iapm | 0.072 ± 0.003 | 0.059 ± 0.003 | 0.064 | 0.305 |
|  | lat Ia | 0.088 ± 0.003 | 0.074 ± 0.004 | 0.087 | 0.338 |
|  | OLF | 0.066 ± 0.002 | 0.059 ± 0.003 | 0.165 | 0.438 |
|  | G | 0.072 ± 0.003 | 0.063 ± 0.004 | 0.300 | 0.509 |
|  | PrCO | 0.073 ± 0.003 | 0.067 ± 0.004 | 0.333 | 0.514 |
|  | area 8A | 0.123 ± 0.005 | 0.152 ± 0.008 | 0.090 | 0.338 |
|  | area 8B | 0.147 ± 0.006 | 0.161 ± 0.007 | 0.508 | 0.653 |
|  | area 9 | 0.120 ± 0.005 | 0.098 ± 0.005 | 0.128 | 0.385 |
|  | area 46d | 0.119 ± 0.005 | 0.102 ± 0.005 | 0.194 | 0.460 |
|  | area 46v/f | 0.132 ± 0.006 | 0.113 ± 0.006 | 0.320 | 0.514 |
|  | area 12r/l | 0.094 ± 0.005 | 0.079 ± 0.003 | 0.189 | 0.460 |
|  | area 45 | 0.106 ± 0.004 | 0.110 ± 0.006 | 0.808 | 0.845 |
|  | area 44 | 0.088 ± 0.004 | 0.079 ± 0.005 | 0.355 | 0.514 |
|  | M1 | 0.170 ± 0.006 | 0.181 ± 0.007 | 0.603 | 0.733 |
|  | PMd | 0.168 ± 0.007 | 0.187 ± 0.007 | 0.365 | 0.514 |
|  | PMv | 0.123 ± 0.005 | 0.129 ± 0.006 | 0.703 | 0.809 |
|  | preSMA | 0.131 ± 0.006 | 0.141 ± 0.006 | 0.692 | 0.809 |
|  | SMA | 0.136 ± 0.007 | 0.145 ± 0.007 | 0.737 | 0.809 |
|  | area 3a/b | 0.150 ± 0.006 | 0.129 ± 0.006 | 0.106 | 0.348 |
|  | areas 1-2 | 0.148 ± 0.006 | 0.168 ± 0.007 | 0.242 | 0.479 |
|  | SII | 0.110 ± 0.004 | 0.120 ± 0.006 | 0.379 | 0.517 |
|  | area 5d | 0.151 ± 0.005 | 0.201 ± 0.006 | **<0.001***** | **0.011*** |
|  | PEa | 0.139 ± 0.005 | 0.182 ± 0.006 | **0.007**** | 0.085 |
|  | AIP | 0.085 ± 0.003 | 0.083 ± 0.004 | 0.725 | 0.809 |
|  | LIP | 0.166 ± 0.006 | 0.184 ± 0.006 | 0.305 | 0.509 |
|  | area 7op | 0.113 ± 0.004 | 0.159 ± 0.006 | **<0.001***** | **0.011*** |
|  | area 31 | 0.127 ± 0.005 | 0.137 ± 0.005 | 0.554 | 0.692 |
|  | area 23 | 0.158 ± 0.006 | 0.172 ± 0.005 | 0.464 | 0.615 |
| **Degree centrality (Dc)** | area 32 | 6.917 ± 0.260 | 6.309 ± 0.371 | 0.442 | 0.560 |
|  | area 25 | 5.332 ± 0.234 | 4.541 ± 0.372 | 0.233 | 0.392 |
|  | area 24a/b | 8.835 ± 0.310 | 6.377 ± 0.457 | **0.009**** | 0.069 |
|  | area 24c | 10.977 ± 0.266 | 9.661 ± 0.434 | **0.044*** | 0.125 |
|  | area 24a/b prime | 9.516 ± 0.339 | 8.725 ± 0.455 | 0.474 | 0.576 |
|  | area 24c prime | 10.788 ± 0.246 | 11.476 ± 0.283 | 0.301 | 0.452 |
|  | area 10 | 8.627 ± 0.285 | 7.034 ± 0.450 | **0.018*** | 0.101 |
|  | area 14 | 6.311 ± 0.251 | 5.186 ± 0.356 | 0.095 | 0.224 |
|  | area 11 | 7.811 ± 0.263 | 7.088 ± 0.488 | 0.326 | 0.474 |
|  | area 13 | 9.362 ± 0.250 | 7.811 ± 0.477 | **0.035*** | 0.120 |
|  | area 12m/o | 7.981 ± 0.283 | 7.283 ± 0.399 | 0.371 | 0.522 |
|  | Iam/Iapm | 5.980 ± 0.267 | 5.096 ± 0.365 | 0.189 | 0.354 |
|  | lat Ia | 7.686 ± 0.282 | 6.463 ± 0.366 | 0.123 | 0.251 |
|  | OLF | 5.508 ± 0.289 | 5.066 ± 0.326 | 0.636 | 0.666 |
|  | G | 5.997 ± 0.249 | 5.514 ± 0.383 | 0.396 | 0.540 |
|  | PrCO | 6.004 ± 0.227 | 5.923 ± 0.331 | 0.881 | 0.901 |
|  | area 8A | 10.872 ± 0.236 | 13.102 ± 0.404 | **0.002**** | **0.020*** |
|  | area 8B | 12.748 ± 0.289 | 13.861 ± 0.307 | 0.235 | 0.392 |
|  | area 9 | 10.467 ± 0.273 | 8.771 ± 0.408 | **0.033*** | 0.120 |
|  | area 46d | 10.340 ± 0.266 | 9.183 ± 0.439 | **0.035*** | 0.120 |
|  | area 46v/f | 11.632 ± 0.310 | 10.461 ± 0.468 | 0.245 | 0.393 |
|  | area 12r/l | 7.884 ± 0.304 | 7.060 ± 0.360 | 0.286 | 0.443 |
|  | area 45 | 9.299 ± 0.227 | 10.356 ± 0.417 | 0.089 | 0.222 |
|  | area 44 | 7.491 ± 0.251 | 6.994 ± 0.398 | 0.486 | 0.576 |
|  | M1 | 13.839 ± 0.211 | 14.324 ± 0.248 | 0.418 | 0.553 |
|  | PMd | 13.975 ± 0.217 | 15.031 ± 0.240 | **0.045*** | 0.125 |
|  | PMv | 10.811 ± 0.252 | 11.720 ± 0.424 | 0.230 | 0.392 |
|  | preSMA | 11.330 ± 0.286 | 13.066 ± 0.285 | **0.035*** | 0.120 |
|  | SMA | 11.619 ± 0.297 | 12.361 ± 0.361 | 0.448 | 0.560 |
|  | area 3a/b | 12.616 ± 0.251 | 11.117 ± 0.337 | **0.016*** | 0.101 |
|  | areas 1-2 | 12.528 ± 0.246 | 13.973 ± 0.308 | **0.037*** | 0.120 |
|  | SII | 9.506 ± 0.252 | 10.771 ± 0.426 | 0.052 | 0.137 |
|  | area 5d | 12.595 ± 0.229 | 15.631 ± 0.233 | **<0.001***** | **<0.001***** |
|  | PEa | 11.429 ± 0.244 | 14.377 ± 0.229 | **<0.001***** | **<0.001***** |
|  | AIP | 7.201 ± 0.262 | 7.619 ± 0.427 | 0.626 | 0.666 |
|  | LIP | 13.875 ± 0.227 | 14.848 ± 0.238 | 0.102 | 0.230 |
|  | area 7op | 9.804 ± 0.258 | 13.618 ± 0.318 | **<0.001***** | **<0.001***** |
|  | area 31 | 10.896 ± 0.250 | 11.884 ± 0.361 | 0.176 | 0.344 |
|  | area 23 | 13.203 ± 0.222 | 14.099 ± 0.223 | 0.109 | 0.233 |
| **Nodal clustering coefficient (NCp)** | area 32 | 0.148 ± 0.003 | 0.147 ± 0.005 | 0.849 | 0.957 |
|  | area 25 | 0.130 ± 0.003 | 0.108 ± 0.005 | **0.001**** | **0.020*** |
|  | area 24a/b | 0.153 ± 0.003 | 0.132 ± 0.006 | **0.005**** | **0.034*** |
|  | area 24c | 0.159 ± 0.003 | 0.162 ± 0.004 | 0.770 | 0.937 |
|  | area 24a/b prime | 0.159 ± 0.003 | 0.173 ± 0.005 | 0.131 | 0.307 |
|  | area 24c prime | 0.176 ± 0.003 | 0.198 ± 0.003 | **0.002**** | **0.022*** |
|  | area 10 | 0.154 ± 0.003 | 0.144 ± 0.005 | 0.144 | 0.308 |
|  | area 14 | 0.137 ± 0.003 | 0.128 ± 0.004 | 0.080 | 0.232 |
|  | area 11 | 0.154 ± 0.003 | 0.158 ± 0.006 | 0.726 | 0.907 |
|  | area 13 | 0.151 ± 0.003 | 0.150 ± 0.005 | 0.890 | 0.975 |
|  | area 12m/o | 0.151 ± 0.003 | 0.150 ± 0.005 | 0.851 | 0.957 |
|  | Iam/Iapm | 0.133 ± 0.003 | 0.132 ± 0.007 | 0.910 | 0.975 |
|  | lat Ia | 0.139 ± 0.003 | 0.133 ± 0.005 | 0.420 | 0.590 |
|  | OLF | 0.128 ± 0.003 | 0.116 ± 0.005 | 0.168 | 0.329 |
|  | G | 0.149 ± 0.004 | 0.138 ± 0.006 | 0.230 | 0.423 |
|  | PrCO | 0.142 ± 0.003 | 0.142 ± 0.006 | 0.943 | 0.986 |
|  | area 8A | 0.170 ± 0.003 | 0.178 ± 0.004 | 0.412 | 0.590 |
|  | area 8B | 0.162 ± 0.002 | 0.176 ± 0.003 | **0.027*** | 0.095 |
|  | area 9 | 0.162 ± 0.003 | 0.171 ± 0.004 | 0.284 | 0.473 |
|  | area 46d | 0.164 ± 0.003 | 0.176 ± 0.005 | 0.243 | 0.423 |
|  | area 46v/f | 0.157 ± 0.002 | 0.165 ± 0.005 | 0.300 | 0.483 |
|  | area 12r/l | 0.152 ± 0.003 | 0.157 ± 0.004 | 0.546 | 0.702 |
|  | area 45 | 0.158 ± 0.003 | 0.164 ± 0.004 | 0.503 | 0.685 |
|  | area 44 | 0.156 ± 0.003 | 0.156 ± 0.005 | 0.969 | 0.991 |
|  | M1 | 0.168 ± 0.002 | 0.185 ± 0.002 | **0.018*** | 0.072 |
|  | PMd | 0.168 ± 0.002 | 0.181 ± 0.003 | 0.087 | 0.232 |
|  | PMv | 0.157 ± 0.002 | 0.171 ± 0.003 | **0.014*** | 0.072 |
|  | preSMA | 0.166 ± 0.002 | 0.184 ± 0.003 | **0.003**** | **0.029*** |
|  | SMA | 0.170 ± 0.003 | 0.195 ± 0.003 | **<0.001***** | **0.002**** |
|  | area 3a/b | 0.165 ± 0.002 | 0.186 ± 0.004 | **<0.001***** | **0.013*** |
|  | areas 1-2 | 0.165 ± 0.002 | 0.179 ± 0.003 | **0.017*** | 0.072 |
|  | SII | 0.159 ± 0.003 | 0.175 ± 0.004 | **0.013*** | 0.072 |
|  | area 5d | 0.173 ± 0.003 | 0.176 ± 0.002 | 0.803 | 0.951 |
|  | PEa | 0.175 ± 0.003 | 0.184 ± 0.003 | 0.353 | 0.530 |
|  | AIP | 0.166 ± 0.003 | 0.183 ± 0.004 | 0.065 | 0.210 |
|  | LIP | 0.165 ± 0.002 | 0.180 ± 0.003 | **0.023*** | 0.085 |
|  | area 7op | 0.175 ± 0.003 | 0.184 ± 0.002 | 0.136 | 0.307 |
|  | area 31 | 0.175 ± 0.003 | 0.188 ± 0.003 | 0.168 | 0.329 |
|  | area 23 | 0.171 ± 0.003 | 0.183 ± 0.003 | 0.084 | 0.232 |

Note: Data were presented as mean ± S.E.M. KO, *n* = 4 giant *ANK2* knockout monkeys, including 2 rhesus and 2 cynomolgus monkeys; WT, *n* = 11 wild-type monkeys, including 5 rhesus and 6 cynomolgus monkeys; FCD, functional connectivity density; Dc, degree centrality; NCp, nodal clustering coefficient.

*, *p* < 0.05; **, *p* < 0.01; ***, *p* < 0.001.

**Table S5 Comparison of the structural and functional MRI parameters in subcortical regions between the giant *ANK2* knockout (KO) and wild-type (WT) monkeys**

| **Parameter** | **Region** | **WT (*n* = 11)** | **KO (*n* = 4)** | ***p* value** | **Corrected *p* value** |
| --- | --- | --- | --- | --- | --- |
| **Myelination** | LVPal | 0.185 ± 0.003 | 0.197 ± 0.010 | 0.108 | 0.418 |
|  | Amy | 0.126 ± 0.003 | 0.137 ± 0.008 | 0.093 | 0.418 |
|  | Pu | 0.199 ± 0.003 | 0.221 ± 0.010 | **0.008***** | 0.117 |
|  | Acb | 0.139 ± 0.002 | 0.150 ± 0.006 | **0.036*** | 0.301 |
|  | Pd | 0.361 ± 0.007 | 0.419 ± 0.017 | **0.001***** | **0.019*** |
|  | Hy | 0.219 ± 0.004 | 0.257 ± 0.009 | **<0.001***** | **0.004**** |
| **Volume** | LVPal | 271.131 ± 13.476 | 274.688 ± 23.973 | 0.922 | 1.000 |
|  | Amy | 262.182 ± 9.485 | 269.016 ± 15.517 | 0.753 | 0.959 |
|  | Pu | 767.722 ± 31.513 | 757.625 ± 50.109 | 0.395 | 0.875 |
|  | Acb | 87.216 ± 3.620 | 87.063 ± 9.263 | 0.759 | 0.959 |
|  | Pd | 218.046 ± 9.303 | 191.125 ± 9.768 | **0.001***** | **0.030*** |
|  | Hy | 159.602 ± 5.907 | 151.000 ± 7.260 | 0.055 | 0.459 |
| **Functional connectivity density (FCD)** | LVPal | 0.102 ± 0.004 | 0.091 ± 0.005 | 0.283 | 0.509 |
|  | Amy | 0.066 ± 0.003 | 0.065 ± 0.004 | 0.827 | 0.846 |
|  | Pu | 0.127 ± 0.005 | 0.099 ± 0.005 | **0.017*** | 0.126 |
|  | Acb | 0.077 ± 0.003 | 0.057 ± 0.003 | **0.008**** | 0.085 |
|  | Pd | 0.055 ± 0.002 | 0.049 ± 0.003 | 0.214 | 0.479 |
|  | Hy | 0.054 ± 0.002 | 0.052 ± 0.003 | 0.791 | 0.845 |
| **Degree centrality (Dc)** | LVPal | 9.033 ± 0.273 | 8.607 ± 0.476 | 0.564 | 0.619 |
|  | Amy | 5.465 ± 0.261 | 5.907 ± 0.431 | 0.523 | 0.603 |
|  | Pu | 11.350 ± 0.268 | 9.277 ± 0.375 | **0.002**** | **0.019*** |
|  | Acb | 6.525 ± 0.289 | 4.762 ± 0.292 | **0.024*** | 0.120 |
|  | Pd | 4.377 ± 0.218 | 3.969 ± 0.329 | 0.546 | 0.614 |
|  | Hy | 4.308 ± 0.239 | 4.352 ± 0.357 | 0.941 | 0.941 |
| **Nodal clustering coefficient (NCp)** | LVPal | 0.155 ± 0.003 | 0.148 ± 0.005 | 0.343 | 0.530 |
|  | Amy | 0.138 ± 0.004 | 0.148 ± 0.006 | 0.245 | 0.423 |
|  | Pu | 0.160 ± 0.003 | 0.161 ± 0.004 | 0.993 | 0.993 |
|  | Acb | 0.136 ± 0.003 | 0.124 ± 0.005 | 0.103 | 0.258 |
|  | Pd | 0.133 ± 0.004 | 0.128 ± 0.007 | 0.535 | 0.702 |
|  | Hy | 0.116 ± 0.004 | 0.139 ± 0.009 | **0.016*** | 0.072 |

Note: Data were presented as mean ± S.E.M. KO, *n* = 4 giant *ANK2* knockout monkeys, including 2 rhesus and 2 cynomolgus monkeys; WT, *n* = 11 wild-type monkeys, including 5 rhesus and 6 cynomolgus monkeys; FCD, functional connectivity density; Dc, degree centrality; NCp, nodal clustering coefficient.

*, *p* < 0.05; **, *p* < 0.01; ***, *p* < 0.001.

**Table S6. Statistical summary and model diagnostics for corrected and nominal MRI/fMRI findings.**

| **Parameter** | **Regions** | ***P*_ShapiroWilk** | ***P*_BrownForsythe** | **Cohens d** | **uncorrected *p* value** | **Corrected *p* value** |
| --- | --- | --- | --- | --- | --- | --- |
| Surface area | area 24a/b | 0.676 | 0.528 | 0.448 | **0.004**** | 0.100 |
| Surface area | SMA | **0.013*** | 0.990 | 0.269 | **0.005**** | 0.100 |
| Surface area | area 46v/f | 0.431 | 0.667 | 0.498 | **0.009**** | 0.120 |
| Surface area | area 8A | 0.407 | 0.590 | 0.446 | **0.017*** | 0.169 |
| Surface area | area 32 | 0.122 | 0.662 | 0.370 | **0.027*** | 0.198 |
| Surface area | area 9 | 0.089 | 0.102 | 0.333 | **0.031*** | 0.198 |
| Surface area | area 24c prime | 0.498 | 0.752 | -0.374 | **0.036*** | 0.200 |
| Surface area | LIP | 0.238 | **0.006**** | 0.346 | **0.045*** | 0.217 |
| Thickness | area 24a/b | 0.402 | 0.964 | 1.652 | **<0.001***** | **<0.001***** |
| Thickness | area 24a/b prime | 0.861 | 0.835 | 1.026 | **<0.001***** | **0.007**** |
| Thickness | area 24c | 0.283 | 0.230 | -1.617 | **0.001**** | **0.007**** |
| Thickness | area 25 | 0.339 | 0.737 | 1.195 | **0.003**** | **0.033*** |
| Thickness | area 32 | 0.769 | 0.704 | 0.822 | **0.019*** | 0.147 |
| Gray matter volume | area 24a/b | 0.867 | 0.603 | 0.651 | **<0.001***** | **0.024*** |
| Gray matter volume | area 32 | 0.930 | 0.335 | 0.419 | **0.006**** | 0.090 |
| myelination in cortex | M1 | 0.744 | 0.262 | -1.425 | **0.036*** | 0.301 |
| myelination in cortex | Iam/Iapm | **0.004**** | 0.798 | -0.888 | **0.040*** | 0.301 |
| myelination in subcortex | Hy | 0.131 | 0.318 | -2.936 | **<0.001***** | **0.004**** |
| myelination in subcortex | Pd | 0.935 | 0.263 | -1.463 | **<0.001***** | **0.019*** |
| myelination in subcortex | Pu | 0.234 | 0.098 | -1.533 | **0.008**** | 0.117 |
| myelination in subcortex | Acb | **0.040*** | 0.173 | -0.853 | **0.036*** | 0.301 |
| subcortex volume | Pd | **0.042*** | 0.838 | 0.579 | **0.001**** | **0.030*** |
| NCp | SMA | 0.211 | 0.706 | -0.787 | **<0.001***** | **0.002**** |
| NCp | area 3a/b^a^ | 0.196 | **0.044*** | -0.968 | **<0.001***** | **0.013*** |
| NCp | area 25 | **<0.001***** | 0.684 | 0.640 | **0.001**** | **0.020*** |
| NCp | area 24c prime | 0.510 | 0.633 | -0.684 | **0.002**** | **0.022*** |
| NCp | preSMA | 0.205 | 0.188 | -0.689 | **0.003**** | **0.029*** |
| NCp | area 24a/b | **0.003**** | 0.797 | 0.671 | **0.005**** | **0.034*** |
| NCp | SII | 0.530 | 0.755 | -0.580 | **0.013*** | 0.072 |
| NCp | PMv | **0.042*** | 0.225 | -0.561 | **0.014*** | 0.072 |
| NCp | Hy | **0.011*** | **0.034*** | -0.493 | **0.016*** | 0.072 |
| NCp | areas 1-2 | 0.215 | 0.843 | -0.595 | **0.017*** | 0.072 |
| NCp | M1 | 0.351 | 0.583 | -0.696 | **0.018*** | 0.072 |
| NCp | LIP | 0.174 | 0.517 | -0.674 | **0.023*** | 0.085 |
| NCp | area 8B | **0.005**** | 0.362 | -0.622 | **0.027*** | 0.095 |
| Dc | area 7op | 0.225 | 0.063 | -1.505 | **<0.001***** | **<0.001***** |
| Dc | area 5d^b^ | 0.546 | **<0.001***** | -1.379 | **<0.001***** | **<0.001***** |
| Dc | PEa^c^ | 0.203 | **0.011*** | -1.205 | **<0.001***** | **<0.001***** |
| Dc | Pu | 0.195 | 0.754 | 0.775 | **0.002**** | **0.019*** |
| Dc | area 8A | 0.382 | 0.944 | -0.887 | **0.002**** | **0.020*** |
| Dc | area 24a/b | 0.721 | 0.215 | 0.778 | **0.009**** | 0.069 |
| Dc | area 3a/b | 0.185 | 0.554 | 0.599 | **0.016*** | 0.101 |
| Dc | area 10 | 0.516 | 0.297 | 0.538 | **0.018*** | 0.101 |
| Dc | Acb | 0.215 | **0.039*** | 0.593 | **0.024*** | 0.120 |
| Dc | area 9 | 0.796 | 0.885 | 0.608 | **0.033*** | 0.120 |
| Dc | area 46d | 0.834 | 0.935 | 0.418 | **0.035*** | 0.120 |
| Dc | preSMA | 0.325 | 0.494 | -0.567 | **0.035*** | 0.120 |
| Dc | area 13 | 0.461 | **0.003**** | 0.565 | **0.035*** | 0.120 |
| Dc | areas 1-2 | 0.099 | 0.174 | -0.589 | **0.037*** | 0.120 |
| Dc | area 24c | 0.549 | 0.772 | 0.472 | **0.044*** | 0.125 |
| Dc | PMd | 0.342 | 0.094 | -0.467 | **0.045*** | 0.125 |
| FCD | area 7op | 0.499 | 0.290 | -1.061 | **<0.001***** | **0.011*** |
| FCD | area 5d | **<0.001***** | 0.058 | -0.934 | **<0.001***** | **0.011*** |
| FCD | PEa | **0.006**** | 0.490 | -0.742 | **0.007**** | 0.085 |
| FCD | Acb | **0.001**** | **0.012*** | 0.685 | **0.008**** | 0.085 |
| FCD | area 24a/b | 0.100 | **0.025*** | 0.761 | **0.010**** | 0.088 |
| FCD | Pu | **<0.001***** | 0.914 | 0.556 | **0.017*** | 0.126 |
| FCD | area 14 | **0.001**** | 0.331 | 0.552 | **0.036*** | 0.234 |
| FCD | area 10 | **0.030*** | 0.084 | 0.515 | **0.046*** | 0.259 |
| intra hemisphere link | par-par | 0.060 | 0.706 | -0.738 | **0.004**** | **0.025*** |
| intra hemisphere link | sub-fro^d^ | 0.950 | **0.014*** | 0.689 | **0.011*** | **0.032*** |
| inter hemisphere link | par-par | 0.755 | 0.307 | -0.596 | **0.025*** | 0.053 |
| inter hemisphere link | sub-fro | 0.651 | 0.730 | 0.641 | **0.026*** | 0.053 |
| inter hemisphere link | sub-sub | 0.976 | 0.136 | 0.491 | **0.026*** | 0.053 |
| Small worldness | global | **0.042*** | 0.161 | 1.379 | **0.007**** | **0.007**** |

^a^ Welch t-test, *p* = 0.074; ^b^ Welch t-test, *p* < 0.001; ^c^ Welch t-test, *p* < 0.001; ^d^ Welch t-test, *p* = 0.072

Note: *P_*ShapiroWilk, p value from the Shapiro-Wilk test of normality; *P*_BrownForsythe, *p* value from the Brown-Forsythe test of homogeneity of variance; FCD, functional connectivity density; Dc, degree centrality; NCp, nodal clustering coefficient.

*, *p* < 0.05; **, *p* < 0.01; ***, *p* < 0.001.

**Table S7 Characteristics and comparison of echocardiography between the giant *ANK2* knockout (KO) and wild-type (WT) monkeys**

| **Variable** | **Unit** | **WT (*n* = 11)** | **KO (*n* = 4)** | ***t* / *U*** | ***p* value** |
| --- | --- | --- | --- | --- | --- |
| RVEDD | mm | 7.23 ± 0.66 | 6.89 ± 1.43 | 20 (*U*) | 0.832 |
| LVEDD | mm | 18.28 ± 1.13 | 19.60 ± 2.90 | 0.524 (*t*) | 0.609 |
| IVSD | mm | 4.38 ± 0.34 | 4.63 ± 0.85 | 0.334 (*t*) | 0.744 |
| LVPWD | mm | 4.71 ± 0.24 | 4.63 ± 0.33 | 0.162 (*t*) | 0.874 |
| LVESD | mm | 12.31 ± 0.88 | 12.93 ± 2.18 | 0.323 (*t*) | 0.752 |
| IVSS | mm | 5.65 ± 0.39 | 5.69 ± 0.85 | 0.054 (*t*) | 0.957 |
| LVPWS | mm | 6.02 ± 0.30 | 5.73 ± 0.65 | 0.452 (*t*) | 0.659 |
| RAD | mm | 13.81 ± 0.72 | 13.14 ± 2.41 | 0.369 (*t*) | 0.718 |
| LAD | mm | 11.75 ± 0.77 | 10.79 ± 1.24 | 0.646 (*t*) | 0.529 |
| AOD | mm | 10.17 ± 0.36 | 10.06 ± 0.93 | 0.140 (*t*) | 0.891 |
| AVV | m/s | 1.13 ± 0.05 | 0.94 ± 0.05 | 2.180 (*t*) | **0.048*** |
| PAD | mm | 9.69 ± 0.30 | 9.56 ± 0.74 | 0.208 (*t*) | 0.838 |
| PVV | m/s | 1.00 ± 0.04 | 1.03 ± 0.10 | 0.452 (*t*) | 0.659 |
| FS | % | 33.00 ± 1.26 | 34.33 ± 1.76 | 0.568 (*t*) | 0.580 |
| EF | % | 64.67 ± 1.93 | 66.42 ± 2.74 | 0.484 (*t*) | 0.637 |
| E-wave velocity | m/s | 87.06 ± 7.19 | 79.50 ± 5.18 | 0.389 (*t*) | 0.704 |
| A-wave velocity | m/s | 86.64 ± 5.28 | 90.25 ± 6.59 | 0.545 (*t*) | 0.595 |
| E/A ratio | % | 1.03 ± 0.09 | 0.88 ± 0.02 | 21 (*U*) | 0.930 |

Note: Data were presented as mean ± S.E.M. KO, *n* = 4 giant *ANK2* knockout monkeys, including 2 rhesus and 2 cynomolgus monkeys; WT, *n* = 11 wild-type monkeys, including 5 rhesus and 6 cynomolgus monkeys. The *p* values were defined by the Student’s *t* tests or the Mann-Whitney U test. *, *p* <0.05; *t:* Student’s *t* statistic value; *U*: Mann-Whitney U statistic value.

RVEDD, right ventricle end-diastolic diameter; LVEDD, left ventricle end-diastolic diameter; IVSD, interventricular septal thickness in diastole; LVPWD, left ventricular posterior wall thickness in diastole; LVESD, left ventricular end- systolic dimension; IVSS, interventricular septal thickness in systole; LVPWS, left ventricular posterior wall thickness in systole; RAD, Right atrial diameter; LAD, left atrial diameter; AOD, aorta diameter; AVV, aortic valve velocity; PAD, Pulmonary artery diameter; PVV, pulmonary valve velocity; FS, fractional shortening; EF, ejection fraction.

**Table S8 Hematological parameters with non-significant difference between the giant *ANK2* knockout (KO) and wild-type (WT) monkeys**

| **Variable** | **Unit** | **WT** | **KO** | ***t* / *U*** | ***p* value** |
| --- | --- | --- | --- | --- | --- |
| **Blood biochemistry test (BCT, WT group, n=78; KO group, *n* = 4)** | | | | | |
| AST | U/L | 63.44 ± 7.95 | 45.10 ± 7.75 | 132.5 (*U*) | 0.628 |
| ALP | U/L | 375.92 ± 17.83 | 271.65 ± 70.11 | 100 (*U*) | 0.243 |
| GGT | U/L | 91.91 ± 5.03 | 81.70 ± 11.46 | 135 (*U*) | 0.668 |
| CRE2 | μmol/L | 117.06 ± 2.76 | 112.05 ± 11.15 | 137 (*U*) | 0.698 |
| CK | U/L | 333.68 ± 36.94 | 348.75 ± 241.83 | 108 (*U*) | 0.318 |
| GLU | mmol/L | 6.20 ± 0.26 | 4.63 ± 0.35 | 1.360 (*t*) | 0.178 |
| **Complete blood count (CBC,** **WT group, n=11; KO group, *n* = 4)** | | | | | |
| WBC | 10^9^/L | 11.17 ± 0.79 | 11.18 ± 0.91 | 0.006 (*t*) | 0.995 |
| Lym# | 10^9^/L | 5.67 ± 0.43 | 5.15 ± 0.32 | 188 (*U*) | 0.806 |
| Mon# | 10^9^/L | 0.63 ± 0.06 | 0.72 ± 0.07 | 0.816 (*t*) | 0.419 |
| Gran# | 10^9^/L | 4.87 ± 0.40 | 5.32 ± 0.85 | 189 (*U*) | 0.825 |
| Lym% | % | 50.99 ± 1.65 | 48.8 ± 3.98 | 0.606 (*t*) | 0.547 |
| Mon% | % | 5.62 ± 0.30 | 6.42 ± 0.49 | 1.358 (*t*) | 0.182 |
| Gran% | % | 43.38 ± 1.62 | 44.78 ± 4.00 | 0.390 (*t*) | 0.699 |
| HGB | g/L | 147.12 ± 1.05 | 148.67 ± 2.10 | 0.717 (*t*) | 0.477 |
| HCT | % | 44.13 ± 0.75 | 45.34 ± 0.73 | 171.5 (*U*) | 0.505 |
| MCH | pg | 24.4 ± 0.17 | 23.92 ± 0.26 | 1.507 (*t*) | 0.139 |
| MCHC | g/L | 329 ± 1.86 | 327.67 ± 3.26 | 0.364 (*t*) | 0.717 |
| RDW | % | 12.3 ± 0.23 | 12.48 ± 0.34 | 171 (*U*) | 0.497 |
| PLT | 10^9^/L | 407.58 ± 16.94 | 417.58 ± 18.42 | 0.330 (*t*) | 0.743 |
| MPV | fL | 9.75 ± 0.21 | 9.83 ± 0.38 | 0.176 (*t*) | 0.861 |
| PDW | % | 15.47 ± 0.10 | 15.33 ± 0.14 | 170.5 (*U*) | 0.487 |
| PCT | % | 0.4 ± 0.02 | 0.40 ± 0.01 | 0.287 (*t*) | 0.776 |

Note: Data were presented as mean ± S.E.M. KO group, *n* = 4 giant ANK2 knockout monkeys, including 2 rhesus and 2 cynomolgus monkeys; WT group for BCT, *n* = 78 wild-type monkeys, including 58 rhesus and 20 cynomolgus monkeys; WT group for CBC, n = 11 wild-type monkeys, including 5 rhesus and 6 cynomolgus monkeys. The *p* values were defined by the Student’s *t* tests or the Mann-Whitney U test. *t:* Student’s *t* statistic value; *U*: Mann-Whitney U statistic value.

In BCT, AST, aspartate amino transferase; ALP, alkaline phosphatase; GGT, γ-glutamyl transferase; CRE2, creatinine; CK, creatine kinase; GLU, blood glucose. In CBC, WBC, white blood cell; Lym#, number of lymphocytes; Mon#, number of monocytes; Gran#, number of granulocytes; Lym%, proportion of lymphocyte; Mon%, proportion of monocytes; Gran%, proportion of granulocytes; HGB, hemoglobulin; HCT, hematocrit; MCH, mean corpuscular hemoglobulin; MCHC, mean corpuscular hemoglobulin concentration; RDW, red blood cell volume distribution width; PLT, platelet; MPV, mean platelet volume; PDW, platelet volume distribution width; PCT, plateletcrit

**Table S9 Significantly altered lipid species in CSF and serum between the giant *ANK2* knockout (KO) and wild-type (WT) monkeys**

| **Lipid species** | **Class** | **Calibrated m/z** | **Fold change** | ***U*** | ***p* value** |
| --- | --- | --- | --- | --- | --- |
| **CSF** |  |  |  |  |  |
| PI(15:0/17:0) | PI | 809.5/241.2 | 6.39 | 1.5 | 0.0010 |
| TG(52:4)-FA16:0 | TG | 872.8/599.5 | 5.31 | 5 | 0.0060 |
| TG(54:3)-FA18:0 | TG | 902.8/601.5 | 5.31 | 5 | 0.0060 |
| TG(54:3)-FA18:2 | TG | 902.8/605.5 | 5.08 | 4 | 0.0040 |
| TG(52:4)-FA18:2 | TG | 872.8/575.5 | 4.96 | 5 | 0.0060 |
| TG(52:3)-FA16:0 | TG | 874.8/601.5 | 4.83 | 5 | 0.0060 |
| TG(52:5)-FA18:2 | TG | 870.8/573.5 | 4.77 | 3 | 0.0023 |
| TG(52:3)-FA18:2 | TG | 874.8/577.5 | 4.56 | 6 | 0.0090 |
| TG(54:5)-FA18:1 | TG | 898.8/599.5 | 4.46 | 3 | 0.0023 |
| TG(54:5)-FA18:2 | TG | 898.8/601.5 | 4.43 | 3 | 0.0023 |
| TG(54:4)-FA18:0 | TG | 900.8/599.5 | 4.37 | 3 | 0.0023 |
| TG(54:4)-FA18:1 | TG | 900.8/601.5 | 4.37 | 5 | 0.0060 |
| TG(54:4)-FA18:2 | TG | 900.8/603.5 | 4.35 | 5 | 0.0060 |
| TG(56:4)-FA20:1 | TG | 928.8/601.5 | 4.35 | 6 | 0.0090 |
| TG(50:4)-FA14:0 | TG | 844.6/599.4 | 4.32 | 4 | 0.0040 |
| TG(52:5)-FA16:1 | TG | 870.8/599.5 | 4.28 | 5 | 0.0060 |
| TG(56:5)-FA18:2 | TG | 926.8/629.5 | 4.26 | 11 | 0.0404 |
| TG(52:5)-FA16:0 | TG | 870.8/597.5 | 4.20 | 3 | 0.0023 |
| TG(52:4)-FA18:3 | TG | 872.8/577.5 | 4.13 | 6 | 0.0090 |
| TG(52:4)-FA18:1 | TG | 872.8/573.5 | 4.13 | 6 | 0.0090 |
| TG(50:2)-FA18:2 | TG | 848.8/551.5 | 4.06 | 5 | 0.0060 |
| TG(52:5)-FA18:3 | TG | 870.8/575.5 | 4.02 | 5 | 0.0060 |
| TG(50:3)-FA16:0 | TG | 846.8/573.5 | 3.83 | 6 | 0.0090 |
| TG(52:6)-FA16:1 | TG | 868.8/597.5 | 3.80 | 1 | 0.0007 |
| TG(52:3)-FA18:0 | TG | 874.8/573.5 | 3.77 | 6 | 0.0090 |
| TG(52:3)-FA18:1 | TG | 874.8/575.5 | 3.61 | 6 | 0.0090 |
| TG(56:4)-FA18:2 | TG | 928.8/631.5 | 3.58 | 9 | 0.0237 |
| TG(52:5)-FA18:1 | TG | 870.8/571.5 | 3.55 | 3 | 0.0023 |
| TG(54:6)-FA18:1 | TG | 896.8/597.5 | 3.34 | 3 | 0.0023 |
| TG(56:5)-FA18:1 | TG | 926.8/627.5 | 3.33 | 8 | 0.0177 |
| TG(54:6)-FA18:3 | TG | 896.8/601.5 | 3.32 | 3 | 0.0023 |
| TG(52:6)-FA18:2 | TG | 868.8/571.5 | 3.30 | 4 | 0.0040 |
| TG(56:5)-FA20:1 | TG | 926.8/599.5 | 3.29 | 7 | 0.0127 |
| TG(56:8)-FA22:6 | TG | 920.8/575.5 | 3.27 | 8 | 0.0177 |
| TG(52:4)-FA16:1 | TG | 872.8/601.5 | 3.27 | 6 | 0.0090 |
| TG(54:3)-FA20:1 | TG | 902.8/575.5 | 3.19 | 6 | 0.0090 |
| TG(52:6)-FA18:3 | TG | 868.8/573.5 | 3.16 | 0 | 0.0003 |
| TG(54:4)-FA18:3 | TG | 900.8/605.5 | 3.16 | 6 | 0.0090 |
| TG(53:4)-FA17:0 | TG | 886.8/599.5 | 3.14 | 9 | 0.0237 |
| TG(54:4)-FA16:0 | TG | 900.8/627.5 | 3.13 | 9 | 0.0237 |
| TG(54:5)-FA18:3 | TG | 898.8/603.5 | 3.11 | 4 | 0.0040 |
| TG(54:2)-FA18:2 | TG | 904.8/607.5 | 3.08 | 5 | 0.0060 |
| TG(50:3)-FA14:0 | TG | 846.8/601.6 | 3.04 | 5 | 0.0060 |
| TG(54:3)-FA16:0 | TG | 902.8/629.5 | 3.01 | 6 | 0.0090 |
| TG(54:6)-FA18:2 | TG | 896.8/599.5 | 2.97 | 3 | 0.0023 |
| TG(52:2)-FA18:2 | TG | 876.8/579.5 | 2.92 | 5 | 0.0060 |
| TG(56:6)-FA18:1 | TG | 924.8/625.5 | 2.91 | 7 | 0.0127 |
| TG(50:2)-FA16:0 | TG | 848.8/575.5 | 2.91 | 6 | 0.0090 |
| TG(56:4)-FA18:1 | TG | 928.8/629.5 | 2.90 | 3 | 0.0023 |
| TG(50:4)-FA18:2 | TG | 844.6/547.3 | 2.87 | 5 | 0.0060 |
| TG(54:5)-FA16:0 | TG | 898.8/625.5 | 2.86 | 5 | 0.0060 |
| TG(54:5)-FA18:0 | TG | 898.8/597.5 | 2.86 | 7 | 0.0127 |
| TG(54:2)-FA18:0 | TG | 904.8/603.5 | 2.86 | 8 | 0.0177 |
| TG(56:3)-FA20:0 | TG | 930.8/601.5 | 2.86 | 11.5 | 0.0419 |
| TG(50:3)-FA18:2 | TG | 846.8/549.5 | 2.81 | 6 | 0.0090 |
| TG(52:2)-FA16:0 | TG | 876.8/603.5 | 2.80 | 6 | 0.0090 |
| TG(50:4)-FA16:0 | TG | 844.6/571.3 | 2.79 | 7 | 0.0127 |
| TG(52:2)-FA18:0 | TG | 876.8/575.5 | 2.79 | 7 | 0.0127 |
| TG(54:3)-FA18:1 | TG | 902.8/603.5 | 2.71 | 8 | 0.0177 |
| TG(54:4)-FA20:2 | TG | 900.8/575.5 | 2.70 | 8 | 0.0177 |
| TG(54:2)-FA18:1 | TG | 904.8/605.5 | 2.54 | 9 | 0.0237 |
| TG(50:5)-FA18:2 | TG | 842.6/545.3 | 2.52 | 6 | 0.0089 |
| TG(53:4)-FA18:2 | TG | 886.8/589.5 | 2.48 | 8 | 0.0177 |
| TG(50:3)-FA18:3 | TG | 846.8/551.5 | 2.47 | 8 | 0.0177 |
| TG(56:7)-FA22:6 | TG | 922.8/577.5 | 2.44 | 8 | 0.0177 |
| TG(54:4)-FA20:1 | TG | 900.8/573.5 | 2.41 | 5 | 0.0055 |
| TG(56:3)-FA18:1 | TG | 930.8/631.5 | 2.40 | 6 | 0.0090 |
| TG(50:5)-FA14:0 | TG | 842.6/597.4 | 2.38 | 9 | 0.0237 |
| TG(52:2)-FA18:1 | TG | 876.8/577.5 | 2.37 | 6 | 0.0090 |
| TG(56:3)-FA20:1 | TG | 930.8/603.5 | 2.35 | 11 | 0.0404 |
| TG(53:3)-FA17:0 | TG | 888.8/601.5 | 2.33 | 3 | 0.0023 |
| TG(54:7)-FA18:2 | TG | 894.8/597.5 | 2.33 | 2 | 0.0013 |
| DG(18:0/20:4) | DG | 662.6/341.3 | 2.32 | 6 | 0.0090 |
| TG(58:8)-FA22:6 | TG | 948.8/603.5 | 2.31 | 6 | 0.0090 |
| TG(54:5)-FA20:4 | TG | 898.8/577.5 | 2.31 | 9 | 0.0237 |
| TG(54:7)-FA18:3 | TG | 894.8/599.5 | 2.30 | 3 | 0.0023 |
| TG(56:7)-FA18:1 | TG | 922.8/623.5 | 2.28 | 11 | 0.0393 |
| TG(56:9)-FA22:6 | TG | 918.8/573.5 | 2.27 | 5 | 0.0058 |
| TG(56:6)-FA22:4 | TG | 924.8/575.5 | 2.25 | 6 | 0.0089 |
| TG(52:8)-FA18:2 | TG | 864.8/567.5 | 2.24 | 7.5 | 0.0135 |
| PE(O-16:0/18:2) | PE-O | 700.5/279.2 | 2.23 | 6 | 0.0090 |
| PE(18:2/20:4) | PE | 762.5/303.2 | 2.22 | 11 | 0.0404 |
| TG(55:4)-FA18:2 | TG | 914.8/617.5 | 2.17 | 9 | 0.0237 |
| PE(18:1/20:1) | PE | 770.6/309.3 | 2.17 | 3 | 0.0023 |
| PE(18:2/18:2) | PE | 738.5/279.2 | 2.16 | 11 | 0.0404 |
| TG(56:2)-FA20:1 | TG | 932.9/605.6 | 2.16 | 6 | 0.0090 |
| PE(18:1/18:2) | PE | 740.5/279.2 | 2.15 | 11 | 0.0404 |
| DG(18:0/22:4) | DG | 690.6/341.3 | 2.14 | 10 | 0.0314 |
| TG(56:6)-FA20:4 | TG | 924.8/603.5 | 2.13 | 10 | 0.0314 |
| PE(16:0/18:2) | PE | 714.5/279.2 | 2.11 | 9 | 0.0237 |
| TG(54:6)-FA22:6 | TG | 896.8/551.5 | 2.10 | 7 | 0.0127 |
| TG(48:2)-FA18:2 | TG | 820.7/523.5 | 2.04 | 5 | 0.0060 |
| TG(54:5)-FA20:3 | TG | 898.8/575.5 | 2.04 | 11 | 0.0404 |
| TG(50:4)-FA18:3 | TG | 844.6/549.3 | 2.03 | 9 | 0.0237 |
| TG(54:3)-FA20:2 | TG | 902.8/577.5 | 2.02 | 10 | 0.0314 |
| TG(53:3)-FA18:2 | TG | 888.8/591.5 | 2.00 | 8 | 0.0177 |
| TG(56:2)-FA20:0 | TG | 932.9/603.6 | 1.97 | 11 | 0.0404 |
| TG(54:2)-FA16:0 | TG | 904.8/631.5 | 1.95 | 10 | 0.0314 |
| TG(56:3)-FA16:0 | TG | 930.8/657.5 | 1.94 | 10 | 0.0314 |
| PE(17:0/18:2) | PE | 728.5/269.2 | 1.82 | 11 | 0.0404 |
| TG(50:1)-FA16:0 | TG | 850.8/577.5 | 1.80 | 6 | 0.0090 |
| TG(58:3)-FA18:1 | TG | 958.9/659.6 | 1.78 | 8 | 0.0177 |
| PE(16:0/18:1) | PE | 716.5/281.2 | 1.74 | 6 | 0.0090 |
| TG(48:3)-FA14:0 | TG | 818.7/573.5 | 1.74 | 10 | 0.0314 |
| TG(50:4)-FA18:1 | TG | 844.6/545.3 | 1.74 | 6 | 0.0090 |
| ChE(22:2) | ChE | 722.7/369.4 | 1.72 | 8 | 0.0177 |
| TG(52:1)-FA16:0 | TG | 878.8/605.5 | 1.72 | 7 | 0.0127 |
| DG(18:2/20:0) | DG | 666.6/337.3 | 1.70 | 11 | 0.0404 |
| PE(18:0/18:1) | PE | 744.6/281.2 | 1.70 | 9 | 0.0237 |
| TG(52:6)-FA16:0 | TG | 868.8/595.5 | 1.69 | 11 | 0.0404 |
| TG(52:3)-FA16:1 | TG | 874.8/603.5 | 1.68 | 6 | 0.0090 |
| TG(50:1)-FA18:1 | TG | 850.8/551.5 | 1.68 | 6 | 0.0090 |
| TG(52:1)-FA18:0 | TG | 878.8/577.5 | 1.63 | 7 | 0.0127 |
| TG(52:3)-FA20:1 | TG | 874.8/547.5 | 1.62 | 8 | 0.0177 |
| TG(54:4)-FA20:3 | TG | 900.8/577.5 | 1.60 | 11 | 0.0404 |
| TG(54:1)-FA18:1 | TG | 906.8/607.5 | 1.60 | 10 | 0.0314 |
| DG(16:0/22:4) | DG | 662.6/313.3 | 1.59 | 7 | 0.0127 |
| DG(18:0/20:0) | DG | 670.6/341.3 | 1.59 | 11 | 0.0404 |
| TG(55:4)-FA18:1 | TG | 914.8/615.5 | 1.57 | 9 | 0.0237 |
| TG(52:1)-FA18:1 | TG | 878.8/579.5 | 1.53 | 7 | 0.0127 |
| TG(50:2)-FA14:0 | TG | 848.8/603.6 | 1.52 | 10 | 0.0314 |
| TG(52:2)-FA16:1 | TG | 876.8/605.5 | 1.52 | 6 | 0.0090 |
| PE(16:0/16:0) | PE | 690.5/255.2 | 1.51 | 10 | 0.0314 |
| PC(15:0/18:3) | PC | 800.5/241.2 | 0.66 | 11 | 0.0404 |
| TG(56:7)-FA16:0 | TG | 922.8/649.5 | 0.60 | 11 | 0.0393 |
| TG(58:6)-FA18:1 | TG | 952.8/653.5 | 0.40 | 11.5 | 0.0428 |
| **Serum** |  |  |  |  |  |
| PG(16:0/16:1) | PG | 719.5/253.2 | 3.23 | 10.5 | 0.0326 |
| TG(52:8)-FA16:1 | TG | 864.8/593.5 | 2.61 | 11.5 | 0.0424 |
| PS(18:0/22:6) | PS | 834.5/327.2 | 1.97 | 7 | 0.0127 |
| PE(P-18:1/20:0) | PE-P | 758.5/369.3 | 1.65 | 10.5 | 0.0338 |
| PC(20:0/16:1) | PC | 846.6/253.2 | 1.63 | 11.5 | 0.0426 |
| MGDG(18:1/18:1) | MGDG | 800.6/339.3 | 1.63 | 8 | 0.0177 |
| DGDG(18:3/20:1) | DGDG | 986.7/367.3 | 0.30 | 10 | 0.0291 |
| ChE(16:0) | ChE | 642.6/369.4 | 0.26 | 3 | 0.0023 |

Note: Fold change > 1.5 indicates up-regulation and < 2/3 indicates down-regulation. The *p* values were determined by the Mann-Whitney U test. *U*: Mann-Whitney U statistic value. KO group, *n* = 4, including 2 rhesus monkeys and 2 cynomolgus monkeys; WT group, *n* = 17, including 11 rhesus monkeys and 6 cynomolgus monkeys.

PI, phosphatidylinositol; TG, triglyceride; PE, phosphatidylethanolamine; DG, diacylglycerols; PC, phosphatidylcholine; PG, phosphatidylglycerol; PS, phosphatidylserine; PE-O, alkyl-phosphatidylethanolamine; PE-P, alkenyl-phosphatidylethanolamine; MGDG, monogalactosyldiacylglycerol; DGDG, digalactosyldiacylglycerol; ChE, cholesterol Ester
